# Electrophysiology of the Facultative Autotrophic Bacterium *Desulfosporosinus orientis*

**DOI:** 10.3389/fbioe.2020.00457

**Published:** 2020-05-19

**Authors:** Valeria Agostino, Annika Lenic, Bettina Bardl, Valentina Rizzotto, An N. T. Phan, Lars M. Blank, Miriam A. Rosenbaum

**Affiliations:** ^1^Institute of Applied Microbiology (iAMB), Aachen Biology and Biotechnology, RWTH Aachen University, Aachen, Germany; ^2^Bio Pilot Plant, Leibniz Institute for Natural Product Research and Infection Biology – Hans Knöll Institute, Jena, Germany; ^3^Institute of Inorganic Chemistry, RWTH Aachen University, Aachen, Germany; ^4^Faculty of Biological Sciences, Friedrich Schiller University Jena, Jena, Germany

**Keywords:** sulfate-reducing bacteria, *Desulfosporosinus*, bioelectrochemical systems, cathode, microbial electrosynthesis, acetogenesis, CO_2_ reduction

## Abstract

Electroautotrophy is a novel and fascinating microbial metabolism, with tremendous potential for CO_2_ storage and valorization into chemicals and materials made thereof. Research attention has been devoted toward the characterization of acetogenic and methanogenic electroautotrophs. In contrast, here we characterize the electrophysiology of a sulfate-reducing bacterium, *Desulfosporosinus orientis*, harboring the Wood-Ljungdahl pathway and, thus, capable of fixing CO_2_ into acetyl-CoA. For most electroautotrophs the mode of electron uptake is still not fully clarified. Our electrochemical experiments at different polarization conditions and Fe^0^ corrosion tests point to a H_2_- mediated electron uptake ability of this strain. This observation is in line with the lack of outer membrane and periplasmic multi-heme *c*-type cytochromes in this bacterium. Maximum planktonic biomass production and a maximum sulfate reduction rate of 2 ± 0.4 mM day^–1^ were obtained with an applied cathode potential of −900 mV vs. Ag/AgCl, resulting in an electron recovery in sulfate reduction of 37 ± 1.4%. Anaerobic sulfate respiration is more thermodynamically favorable than acetogenesis. Nevertheless, *D. orientis* strains adapted to sulfate-limiting conditions, could be tuned to electrosynthetic production of up to 8 mM of acetate, which compares well with other electroacetogens. The yield per biomass was very similar to H_2_/CO_2_ based acetogenesis. Acetate bioelectrosynthesis was confirmed through stable isotope labeling experiments with Na-H^13^CO_3_. Our results highlight a great influence of the CO_2_ feeding strategy and start-up H_2_ level in the catholyte on planktonic biomass growth and acetate production. In serum bottles experiments, *D. orientis* also generated butyrate, which makes *D. orientis* even more attractive for bioelectrosynthesis application. A further optimization of these physiological pathways is needed to obtain electrosynthetic butyrate production in *D. orientis* biocathodes. This study expands the diversity of facultative autotrophs able to perform H_2_-mediated extracellular electron uptake in Bioelectrochemical Systems (BES). We characterized a sulfate-reducing and acetogenic bacterium, *D. orientis*, able to naturally produce acetate and butyrate from CO_2_ and H_2_. For any future bioprocess, the exploitation of planktonic growing electroautotrophs with H_2_-mediated electron uptake would allow for a better use of the entire liquid volume of the cathodic reactor and, thus, higher productivities and product yields from CO_2_-rich waste gas streams.

## Introduction

Microbial Electrochemical Technologies (MET) represent an innovative biotechnological solution for CO_2_-valorisation and surplus electricity storage (Zhang and Tremblay, 2018). In reductive bioelectrochemical processes electrical energy is used to accomplish cathodic fixation of CO_2_-rich gas streams into valuable products (Bajracharya et al., 2017). This Microbial Electrosynthesis (MES) is based on the special metabolic ability of electroautotrophic microorganisms: they acquire energy by taking up electrons directly or indirectly from electrodes, while using CO_2_ as inorganic carbon source and terminal electron acceptor (Nevin et al., 2010).

In the last decade, several acetogens (e.g., *Sporomusa ovata, Clostridium ljungdahlii, Morella thermoacetica*, and others), methanogens (*Methanococcus maripaludis, Methanobacterium* IM1, and others) and photoautotrophic iron oxidizers (*Rhodopseudomonas palustris* TIE-1), which all harbor this peculiar metabolic feature, have been discovered and characterized (Aryal et al., 2017; Karthikeyan et al., 2019; Rowe et al., 2019). However, electroautotrophy is still widely considered a black box, since the molecular mechanisms beyond this extracellular electron uptake are not completely understood and elucidated for the different cathodically active microorganisms. In anodophilic electroactive species like *Geobacter* and *Shewanella*, a direct extracellular electron transfer occurs via *c*-type outer membrane multiheme cytochromes (c-OMCs) and conductive nanowires (Pentassuglia et al., 2018). To the best of our knowledge, up-to now, c-OMCs or similar proteins have not been characterized in the acetogenic genera *Clostridium, Sporomusa, and Moorella.* The oxidative pathway of acetogens is rather based on the cytoplasmatic electron-bifurcating [FeFe] hydrogenase complex, that coupled Ferrodoxin and NAD(P) reduction with H_2_ oxidation. These bifurcating-hydrogenases contribute to the energy conservation pathway of aceteogens, providing reduced Ferrodixin for the Rnf complex or the Ech complex, in which exergonic electron flow is coupled to vectorial ion transport out of the cell. This chemiosmotic gradient is harnessed then by the ATP synthase for ATP production (Buckel and Thauer, 2018). Consequently, a H_2_-mediated electron uptake via hydrogenases is envisaged for the majority of acetogens in BES. In a cathodic environment, hydrogen availability might be strongly enhanced because it is *in situ* produced as chemically dissolved H_2_ (i.e., does not have to be dissolved from a bulk gas phase) and can be utilized before it comes out of solution. This raises the hypothesis that every hydrogenotrophic acetogens would be able to grow at H_2_-evolving cathodes and perform electrosynthesis. However, the capacity of oxidizing H_2_ for the reduction of CO_2_ via the Wood-Ljungdahl pathway is not exclusively sufficient for an acetogenic species to be able to drive MES (Aryal et al., 2017). The work of Aryal et al. (2017) screened different hydrogenotrophic *Sporomusa* species for acetate production in H_2_CO_2_ fermentation flasks and in H_2_-evolving cathodes. The results showed that not all species were able to perform MES and that a more efficient metabolism in H_2_CO_2_-fed flasks does not necessarily translate into better electrosynthetic performance (Aryal et al., 2017).

Different is the situation for extracellular electron uptake in methanogenic biocatalysts, since some of them (*Methanosarcinales)* harbor not only hydrogenases but also c-OMCs (Yee and Rotaru, 2020). Very recently, a hydrogensase-independent but cell-associated electron uptake feature was reported for *Methanosarcina barkeri* (Rowe et al., 2019). However, in another *Methanosarcinales* member (*Methanosarcina mazei*), the inactivation of c-OMCs did not impact its ability to retrieve extracellular electrons from an electrode (Yee and Rotaru, 2020).

Beyond acetogens and methanogens, other groups of prokaryotes, including iron-reducing bacteria, nitrate-reducing bacteria, and sulfate-reducing bacteria (SRB) have shown electroautotrophic ability, while using terminal acceptors different than CO_2_ for their respiration (e.g., heavy metals, nitrate, sulfate) (Logan et al., 2019). Consequently, electroautotrophs can be exploited not only for MES, but also for bioremediation applications.

As we recently reviewed, autotrophic SRB-based biocathodes represent a very promising strategy for sustainable biotechnological productions and environmental engineering strategies (Agostino and Rosenbaum, 2018). However, the electrophysiology of these biocatalysts is still quite unexplored. The most well studied electroautotrophic SRB is the Fe^0^-corroding gram negative Deltaproteobacterium *Desulfopila corrodens* strain IS4, able to both perform bioelectrochemical sulfate reduction and H_2_ production (Beese-Vasbender et al., 2015; Deutzmann and Spormann, 2017). Zaybak et al. (2018) have recently discovered electroautotrophy and electrosynthetic acetate production in another gram negative sulfate-reducing Deltaproteobacterium, *Desulfobacterium autotrophicum* HRM2, able to fix CO_2_ through the Wood-Ljungdahl pathway.

In a screening for new electroautotrophic candidates, we have identified four possible electroautotrophs: two sulfur-oxidizing bacteria, *Thiobacillus denitrificans* and *Sulfurimonas denitrificans*, and two SRB, *Desulfosporosinus orientis* and *Desulfovibrio piger* (deCamposRodrigues and Rosenbaum, 2014). Among them, *D. orientis* was the best performing strain in terms of current consumption. Furthermore, *D. orientis* possesses interesting metabolic features and its genome is fully sequenced and annotated (Pester et al., 2012). It was isolated from Singapore’s soil in 1959, is strictly anaerobic, and belongs to the Clostridia class (even if it stains gram negative) (Adams and Postgate, 1959; Campbell and Postgate, 1965). It is able to grow chemolithoautotrophically on CO or CO_2_ and H_2_ plus sulfate, sulfite and thiosulfate, employing the Wood-Ljungdahl pathway (Klemps et al., 1985; Cypionka and Pfennig, 1986). During electron acceptor limitation conditions, it produces acetate and small amount of butyrate (Cypionka and Pfennig, 1986). Furthermore, it can utilize a wide array of organic carbon sources, ranging from short and medium chain fatty acids to alcohols and aromatic compounds (Klemps et al., 1985; Robertson et al., 2001).

Consequently, we decided to deeper characterize the electrophysiology of *D. orientis*. To possibly understand the mechanisms of extracellular electron uptake (EEU), we studied *D. orientis’* electroactivity at different applied cathodic potentials (E_cath_) and its ability of using metallic iron (Fe^0^) as extracellular electron donor. We evaluated its bioelectrochemical sulfate removal performances under different polarization conditions. Finally, we investigated *D. orientis’* capacity for acetate bioelectrosynthesis. To this aim, Adaptive Laboratory Evolution (ALE) in sulfate-limiting conditions was employed to push the microbial pathways to acetate production.

## Materials and Methods

### Strain and Culture Conditions

*Desulfosporosinus orientis* DSMZ 765 was purchased from the Deutsche Sammlung Mikroorganismen und Zellkulturen (DSMZ). *D. orientis* was routinely maintained in 50 mL DSMZ 63 medium modified as follow (per liter): NH_4_Cl 1.0 g, CaCl_2_ × 2H_2_O 0.1 g, K_2_HPO_4_ 0.5 g, MgSO_4_ × 7H_2_O 0.3 g, Na_2_SO_4_ 2.6 g, D-sodium lactate 5.6 g, yeast extract 1.0 g, resazurin 0.5 mg, L-cysteine-hydrochloride 0.5 g or 0.3 g, NaHCO_3_ 1 g, vitamin solution #141 DSMZ 5.0 ml and trace solution SL-6 plus iron (0.3 g/L stock) 5 mL (deCamposRodrigues and Rosenbaum, 2014). Heterotrophic cultivation was performed in 250 mL serum bottles (Diagonal GmbH & Co KG, Münster, Germany) with N_2_ atmosphere, statically incubated at 30°C. Autotrophic cultivation of *D. orientis* was performed in 650 mL serum bottles (Gerresheimer, Düsseldorf, Germany) with 50 mL modified DSMZ 63 medium (without lactate and yeast extract) and H_2_:CO_2_ (80:20) atmosphere (1 atm). The serum bottles were incubated at 30°C horizontally in order to increase the gas-liquid contact surface for a better gas solubilization.

### Corrosion Experiments

Corrosion experiments were performed in 650 mL serum bottles with 50 mL of modified DSMZ 63 medium (without cysteine, lactate and yeast extract), 5 g of Fe^0^ granules (1–2 mm-99.98%, Alfa Aesar, Germany) and N_2_/CO_2_ (80:20) atmosphere (1 atm). The experiments were inoculated with *D. orientis* pre-culture adapted to autotrophic growth without cysteine (10% v/v). Two abiotic controls with and without the addition of 18 mM of Na-sulfide and a biotic control without Fe^0^ were performed. In addition, normal autotrophic growth with H_2_/CO_2_ (80:20) was monitored. All experiments were performed in triplicate and statically incubated horizontally at 30°C. Samples were collected every 3 days for H_2_ headspace analysis, acetate and sulfate quantification.

### Adaptive Laboratory Evolution Experiments

Adaptive Laboratory Evolution experiments were performed in the two different sulfate-limiting conditions: 50 and 25% of the optimal sulfate concentration of modified DSMZ 63 medium (18.2 mM), corresponding to 9.1 mM and 4.6 mM, respectively. The ALE experiments were completed after a period of 6 months, for a total of 18 culture transfers. The experiments were performed in 650 mL serum bottles with a H_2_:CO_2_ (80:20) atmosphere using 3.2 mM cysteine for the first four culture transfers and then 1.9 mM cysteine. 10%v/v of stationary phase cultures were used as inoculum for the culture transfers. The performances of the 3rd and 17th culture transfer were characterized in triplicates using late-exponential phase cultures as inoculum.

### Bioelectrochemical Systems Reactors and Electrochemical Procedures

Bioelectrochemical systems experiments were performed in bench-top double chambers H-type reactors, separated by a cation exchange membrane (CMI-7000S, Membranes International, United States) and containing 400 mL working liquid volume (Supplementary Figure S1B). All BES experiments were performed in 3-electrode configuration, applying a fixed cathodic potential or current through the use of a multi-channel VMP3 potentiostat (BioLogic). Cyclic Voltammetry (CV) were recorded from −300 mV to −1000 mV vs. Ag/AgCl_(sat. KCl)_, at 2 mV s^–1^ scan rate. Measurements were performed in biotic (end of the test) and abiotic conditions (before inoculation). EDM-3 graphite electrodes (Novotec GmbH, Germany) were used, with a rectangular shaped anode (counter electrode, area 41.6 cm^2^) and a comb shaped cathode (working electrode, area 156.5 cm^2^). The reference electrode [Ag/AgCl_(sat. KCl)_] was placed in the cathodic chamber and all potentials are reported against this reference.

Both chambers were filled with modified DSMZ 63 medium (without lactate and yeast extract). After overnight flushing of the reactors with N_2,_ Na-bicarbonate and vitamin solution from anaerobic stock solutions were added. In case of labeling experiment, Na-H^13^CO_3_ was used. The final electrolyte pH was in the range of 6.8–7. The gas feeding of both chambers was switched to a mixture of N_2_:CO_2_ (80:20) at a flow rate of 50 ml min^–1^ (or 15 mL min^–1^ during labeling experiments), before inoculation. In the cathodic chamber, the N_2_:CO_2_ (80:20) feeding served as carbon source, while in the anodic chamber it aimed to sparge out the likely abiotically formed oxygen and prevent membrane cross over to the cathode chamber.

All reactor experiments were inoculated with late exponential phase autotrophic pre-cultures (10% volume of the catholyte). The catholyte was agitated at 200 rpm with a magnetic stir bar and the temperature was controlled at 30°C with an integrated water jacket and the use of a recirculation water bath (Julabo Paratherm U1M, Julabo GmbH, Seelbach, Germany).

For a global overview of the distribution of the ingoing and outcoming charges (Q) in *D. orientis’* biocathodes, the charge balance was calculated according to the following equation:

Q+cathodeQ=cysteineQ+sulfate⁢redQ+biomassQorganic⁢acids

Faraday’s law (Q = z × n × F; with z, number of electrons per molecule; n, moles; F, Faraday constant) was used to calculate the charge input in the cysteine and the charge recovered in sulfate reduction, biomass production and organic acids production. The charge input provided by the cathodic current was calculated via the integral of the recorded current over time.

### Scanning Electron Microscopy

Prior to SEM imaging, pieces of the graphite electrode were broken off and washed in distilled sterile water and fixed in a solution of 2.5% glutaraldehyde in PBS, at 4°C overnight. Then the samples were dehydrated by an ethanol series and dried with hexamethyldisilazane (HDMS) (Margaria et al., 2017). The fixed samples were sputtered with a 20 nm gold layer and examined with a SEM at 5–10 kV on a Zeiss DSM 982 Gemini microscope (Zeiss, Germany).

### Analytical Procedures

Quantification of organic acids and sulfate was performed by HPLC using a Beckman System Gold 126 Solvent Module coupled with a System Gold 166 UV-detector (Gold HPLC System, Beckman Coulter, Brea, CA, United States) at 210 and 280 nm, respectively. For organic acids, the column (Metab AAC, ISERA GmbH, Düren, Germany) was eluted isocratically with 5 mM H_2_SO_4_ at flow rate of 0.6 mL min^–1^ and an oven temperature of 40°C. Prior to every measurement, the supernatant samples were centrifuged (Heraus Pico17 microCentrifuge, Thermo Scientific, Germany) for 5 min at 13000 rpm and transferred to HPLC vials. Sulfate quantification was performed with a NUCLEOSIL^®^ Anion II column (Macherey-Nagel, Düren, Germany) packed with NUCLEOSIL^®^ silica gel base material, particle size 10 micron, pore size 300 Å and strongly basic anion exchange capacity of 50 μeq/g. The column was eluted isocratically with 2 mM potassium hydrogen phthalate (pH 5.7) at flow rate of 1.5 mL min^–1^ and with a column oven temperature of 20°C. All supernatant samples were previously diluted with the eluent (1:1), centrifuged (Heraus Pico17 microCentrifuge, Thermo Scientific, Germany) for 5 min at 13000 rpm and filtered through 0.2 μm filters (Rotilabo^®^ syringe filter, CA 0.2 μm, Ø 15 mm).

Quantification of cysteine was performed using reversed-phase UHPLC (X-LC Jasco, Tokyo, Japan) equipped with fluorescence detector (Extinction 340 nm + Emission 455 nm). A Waters XBridge BEH C18 XP column (Waters, Milford, MA, United States) at 25°C and a gradient method were employed. Mobile Phase A and B consisted of 25 mM Na_2_HPO_4_/NaH_2_PO_4_ (pH 7.2) + tetrahydrofuran (95:5, v/v) and 25 mM Na_2_HPO_4_/NaH_2_PO_4_ (pH 7.2) + acetonitrile + methanol (50:15:35, v/v/v), respectively. Prior to quantification, all samples were diluted 1:10 with double-distilled water and a thiols reducing agent tris(2-carboxyethyl) phosphine (10 μL sample, 80 μL water and 10 μL TCEP). Derivatization of reduced samples was performed with *ortho*-phthalaldehyde after alkylation of free sulfhydryl groups with iodoacetic acid (20 μL/50 μL sample).

Off-line H_2_ analysis of serum bottles and reactor headspace was performed with a Multiple Gas Analyzer coupled with Helium Ionization Detector (HID) and Thermal Conductivity Detector (TCD) (SRI 8610C, SRI Instruments, Bad Honnef, Germany). Gas separation was carried out with Mole Sieve 5 Å, 13 × 7 and HayeSep D columns, at 53°C for 10 min. Helium was used as carrier gas, with a pressure of 20 psi. A 2.5 mL gas tight syringe (Hamilton^®^, CS Chromatographie, Germany) was used for sampling.

### Isotopomer Analysis

In order to analyze the isotopic ratio of the extracellular acetate produced by *D. orientis* with GC-MS/MS, a derivatization method via alkylation to propyl-acetate with methyl chloroformate and an extraction with methyl tert-butyl ether were employed (Tumanov et al., 2016). A Trace GC Ultra – TSQ 8000 Triple Quadrupole (Thermo Fisher Scientific, Waltham, MA, United States) with a VF-5 ms + 10 m EZ-Guard column (30 m × 0.25 mm × 0.25 μm, Agilent technologies, Santa Clara, CA, United States) was utilized. Samples (1.0 μL) were injected using split mode (1:50) and an inlet temperature of 200°C. The GC gas carrier was Helium at 1.0 mL min^–1^. The GC oven temperature profile was of 1 min at 45°C, followed by a first ramp 25°C min^–1^ to 60°C and a second ramp of 50°C min^–1^ to 190°C, which was then held for 2 min. The temperature of MS transfer line and ion source were set to 280 and 300°C, respectively. The atom percent ^13^C of derivatized acetate was calculated through the following equation (Hayes, 2004):

C%13=[C13/(C12+C13)]⁢100

## Results

### *D. orientis’* Electroactivity and Corrosion Ability

Since hydrogen is a critical reaction partner in our system, we first evaluated the starting potential of H_2_ evolution at our BES cathode. A duplicate abiotic test at different applied cathodic potentials (E_cath_) was performed, starting from −600 mV vs. Ag/AgCl_sat. KCl_ (all potentials are reported against this reference) and going more negative (Supplementary Figure S1A). The theoretical H_2_ evolution potential in neutral pH conditions is −610 mV and, indeed, at E_cath_ of −600 mV no H_2_ was detected in the cathodic headspace. H_2_ evolution was observed at potentials of −800 mV and lower.

**FIGURE 1:**
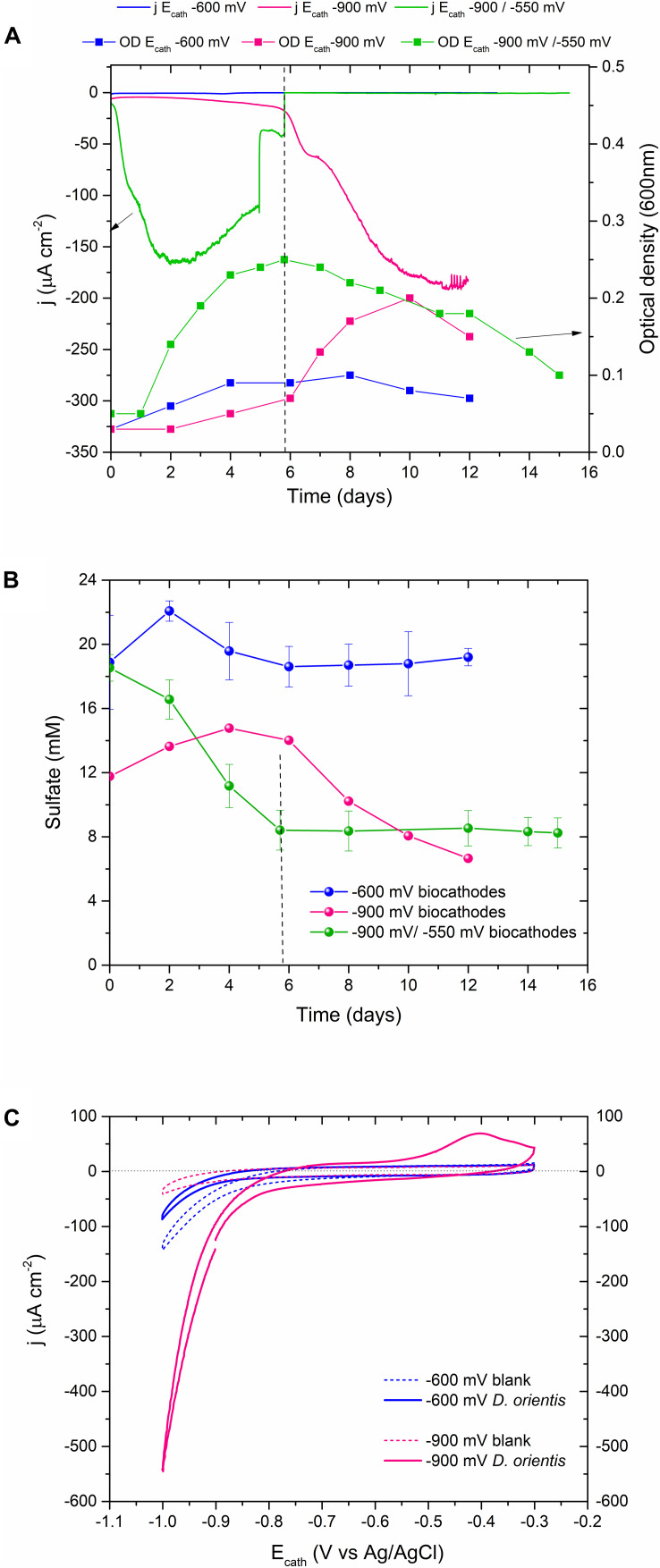
Performances of *Desulfosporosinus orientis* biocathodes at different E_cath_. **(A)** Current density and growth profile. For clarity only one replicate is shown, for all data see Supplementary Figure S2; **(B)** Sulfate quantification in the catholyte; the data represent the average of all conducted replicates and standard deviation. **(C)** Cyclic Voltammetry analysis in blank (before inoculation) and after biotic polarizations (end of the test). Vertical dashed lines indicate the E_cath_ switch time point.

To investigate the electroactivity of *D. orientis*, H-type BES reactors were operated under different cathodic polarization conditions. The first experiment was conducted H_2_-free at an E_cath_ of −600 mV, and the second at −900 mV, where *in-situ* electrochemical H_2_ production occurred. Finally, to boost the initial growth of *D. orientis* and then test its ability to directly use the cathode as electron donor, a third test with a start-up phase of −900 mV and an electrocatalytic phase of −550 mV was carried out. Growth profiles and the current density (j) data are shown in Figure 1A (with replicated data in Supplementary Figure S2). High negative current was only observed in *D. orientis* biocathodes poised at −900 mV, with a maximum current density (j_max_) of −190 μA cm^–2^. The *D. orientis* growth curve followed the current trend: in both −900 mV biocathodes, the exponential growth phase corresponded to the steep increment in current consumption (OD_max_ 0.21 and 0.24). On the contrary, at −550 and −600 mV no planktonic growth was detected (OD_max_ 0.1 and 0.09). These findings suggest an inability of the strain to directly use the energy from the cathodic electrode to maintain its growth. Sulfate quantification data confirmed these results: sulfate removal occurred only when *D. orientis’* biocathodes were poised at −900 mV (Figure 1B).

Cyclic Voltammetry outcomes supported chronoamperometric results, *D. orientis’* growth profile and sulfate data (Figure 1C). The voltammograms of the −900 mV biocathodes showed a reduced overpotential for H_2_ evolution reaction and the maximum cathodic current was much higher, compared to blank analyses. Biotic CV exhibited an anodic wave starting at potentials above −600 mV, with a visible oxidation peak centered at −420 mV. This peak can be related to the oxidation of sulfur species present in the catholyte (Sun et al., 2009). On the contrary, the CV analysis of the −600 mV biocathodes exhibited no different electroactivity compared to blank analyses. At the end of the experiment, graphite pieces of the −600 and −900 mV biocathodes were fixed and analyzed with SEM to investigate *D. orientis* colonization of the electrode surface (Supplementary Figure S2D–E). The graphite surface was not covered by a biofilm and no visible extracellular polymeric substance was observed. Few microbial cells were found only on the −900 mV graphite electrodes.

In order to investigate *D. orientis’* ability of extracellular electron uptake using an inorganic insoluble electron donor, corrosion tests in serum bottle with Fe^0^ granules and N_2_/CO_2_ gas phase were performed (Supplementary Material Section 1). But also here no microbial growth or biological activity beyond abiotic corrosive hydrogen production and consumption was detected (Supplementary Figure S3). In contrast, autotrophic control cultures with H_2_/CO_2_ grew well, readily reduced sulfate and produced acetate (Supplementary Figure S3C).

### Effect of Cathodic Potentials on Bioelectrochemical Sulfate Removal

**FIGURE 2:**
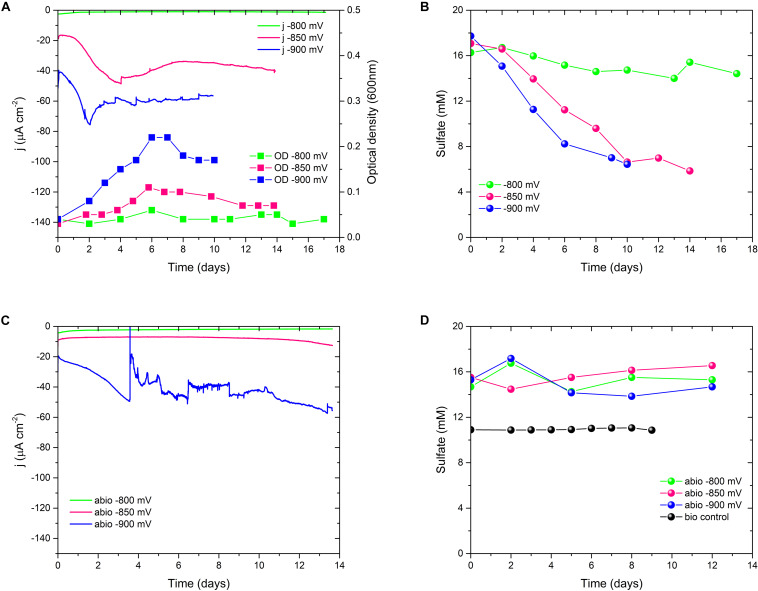
Sulfate reduction performances of *D. orientis* biocathodes and abiotic controls at different E_cath_. **(A)** Current density and *D. orientis* growth profile and **(B)** biotic sulfate reduction trend; **(C)** abiotic current density and **(D)** abiotic and biotic control sulfate reduction profiles. For clarity of data, only one replicate is shown, further replicate data are provided in Supplementary Figure S4.

To investigate the biolectrochemical sulfate removal of *D. orientis* in autotrophic conditions, the biocathode performance was investigated with different E_cath_ ranging from −800 to −900 mV. Those potentials where chosen on basis of biotic CV analysis to find the best E_cath_ in term of sulfate removal and BES coulombic efficiency. Abiotic cathodes with the same polarization conditions and biotic cathodes without potentiostatic control served as controls. Figures 2A,B show the changes of current consumption, *D. orientis* growth and sulfate removal as a function of time, while Table 1 reports a summary of biocathodic/cathodic performances, including coulombic efficiency data. −900 mV biocathodes exhibited the best performances in term of biomass growth (OD_max_ = 0.21 ± 0.02), current density (j_max_ = −75 ± 1 μA cm^–2^), and 10-days sulfate removal (61 ± 2%). However, the total coulombic efficiency of these biocathodes accounted only for 44 ± 0.6%. −800 mV biocathodes resulted, on the contrary, in higher total coulombic efficiency (77 ± 39%) and electron recovery in sulfate reduction (58 ± 25%). However, the cathodic activity and the 10-days sulfate reduction in these −800 mV biocathodes was very low (8 ± 7% sulfate reduction) (see Supplementary Table S1 for statistical analysis of −900 mV vs. −800 mV performances). The control reactors showed no sulfate reduction (Figure 2D), confirming *D. orientis’* involvement in sulfate removal. −900 mV abiotic cathodes exhibited a negative current over time, pointing to another abiotic reduction reaction governing the system at these polarization conditions, most probably abiotic H_2_ evolution (Figures 2C and Supplementary Figure S4E). These findings could explain the low total CE of −900 mV biocathodes, as most likely the unassigned charges are going into abiotic H_2_ production.

**TABLE 1:** Summary of *D. orientis* biocathodes in optimal sulfate concentration conditions.

**Experiment**	**10-days Sulfate removal (%)**	**Max. sulfate reduction rate (SRR) (mM day**^–^**^1^)**	**CE_sulfate_ (%)**	**Tot. CE (%)**	**j_max_ (μA cm**^–^**^2^)**	**Titer acetate (mM)**	**ΔCys (mM)**	**Max. Biomass (mg/L^−1^)**
−900 mV biocathodes 12 days (3.2 mM cys input)	51*	1.9*	22*	34*	−148*	2.4*	2.6*	50*
−900 mV biocathodes 10 days	61 ± 2	2 ± 0.4	37 ± 1.4	44 ± 0.6	−75 ± 1	1 ± 0.3	0.9 ± 0.2	47.3 ± 4.7
−850 mV biocathodes 14 days	54*	1.5*	34*	37*	−60*	0.6*	1.1*	23.0*
−800 mV biocathodes 17 days	8 ± 7	0.8 ± 0.2	58 ± 25	77 ± 39	−5.3 ± 4.9	0.4 ± 0.3	1.1 ± 0.2	7.8 ± 1.5
Abiotic −900 mV	0.1*	–	–	–	−74.5*	–	–	–
Abiotic −850 mV	−0.1#	–	–	–	−12.7#	–	–	–
Abiotic −800 mV	−0.1#	–	–	–	−2.5#	–	–	–
Controls, no potential applied	0.6*	–	–	–	–	0.2*	0*	–

The data represent the average of triplicate or duplicate* reactors and where possible the standard deviation. See Supplementary Table S1 in the Supplementary Material for statistical analysis of relevant comparisons. *duplicate, #single, 1.9 mM cysteine input unless otherwise specified.

**FIGURE 3:**
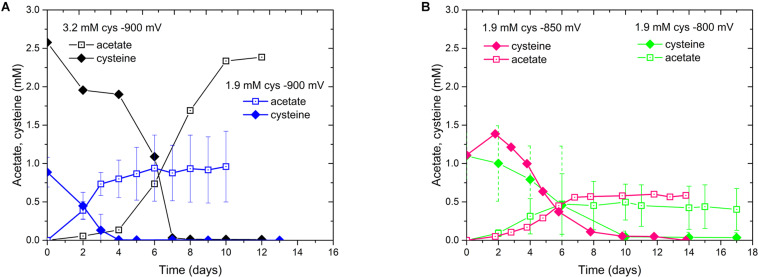
Cysteine consumption and acetate production profiles of *D. orientis* biocathodes at different E_cath_. **(A)** –900 mV with 3.2 and 1.9 mM cysteine input; **(B)** 1.9 mM cysteine input at –850 mV and –800 mV. The data represent the average of triplicate or duplicate reactors and the error bars show standard deviations.

We also noticed the production of acetate in these experiments. Since some bacteria of the class of Clostridia are able to use amino acids as carbon and energy source (Fonknechten et al., 2010), we evaluated the influence of cysteine, which is used as a reducing agent in our media, on our biocathode performance (Supplementary Material Section 2). In the *D. orientis* genome, two genes coding for the key enzyme of the cysteine degradation pathway, a carbon-sulfur lyase, are present (Desor_0514 and Desor_2544). When comparing two different concentrations of initial cysteine input at the different E_cath_, indeed, the final titer of acetate was dependent on the input of cysteine, as well as on the E_cath_ (Figure 3). In addition, the final acetate titer was never higher than the input cysteine concentration (Table 1).

### Adaptive Laboratory Evolution in Sulfate-Limiting Conditions

Anaerobic sulfate respiration is energetically more favorable than acetogenesis (Conrad et al., 1986):

4H+22HCO+3-H→+CHCOO3+-4HO2   ΔG=0′-104.5kJ

2H+2SO+42-H→+HS+-4HO2   ΔG=0′-152.2kJ

**FIGURE 4:**
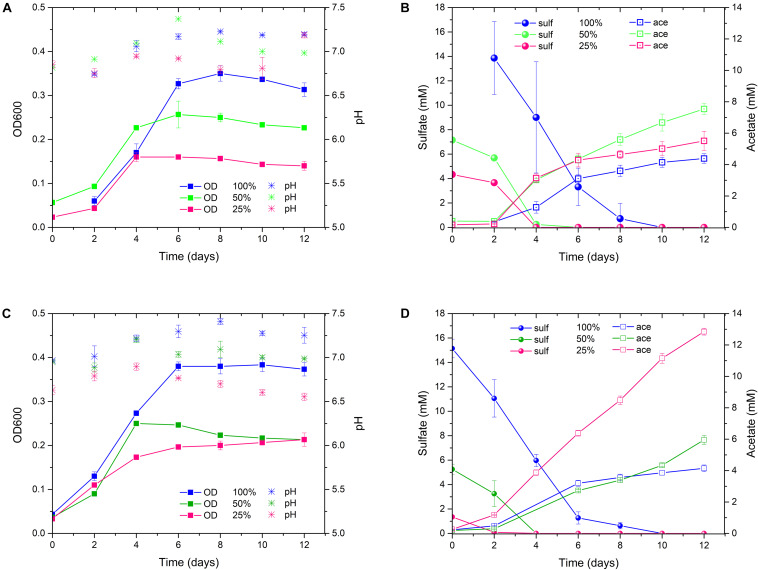
*D. orientis* ALE experiment in sulfate-limiting, autotrophic growth conditions with H_2_/CO_2_. **(A)** pH and OD profiles, **(B)** sulfate reduction and acetate production trends of 3rd culture transfer; **(C)** pH and OD profiles, **(D)** sulfate reduction and acetate production trends of 17th culture transfer. The data represent the average of triplicate cultures and the error bars show standard deviation. 100% = 18 mM sulfate; 50% = 9 mM sulfate; 25% = 4.5 mM sulfate.

In order to foster the acetogenesis at the expense of sulfate respiration, ALE of *D. orientis* in sulfate limiting, autotrophic conditions was performed. Two different stress cultivation conditions were employed: 50 and 25% of the optimal sulfate concentration (18 mM = 100%) recommended for *D. orientis* (deCamposRodrigues and Rosenbaum, 2014). A detailed characterization of the evolved strains was performed after the 3rd and 17th culture transfer (Figure 4, Table 2, and Supplementary Table S2). After three culture transfers, both the 50% and the 25% strains outperformed the 100% strain in term of final acetate titer (*P*-values = 0.0001 and = 0.0174) and yield per maximum biomass (*P*-values = 0.0008 and = 0.0015). In all strains, the maximum acetate productivity was similar (difference not statistically significant) and was reached during the exponential growth phase, for then decreasing during stationary phase. In both sulfate limiting conditions, lower cell growth compared to the optimal cultivation conditions was observed and sulfate was depleted after 4 days of cultivation (Figure 4B).

**TABLE 2:** Performances of *D. orientis* strains adapted to sulfate-limiting growth conditions (100% = 18 mM sulfate; 50% = 9 mM sulfate; 25% = 4.5 mM sulfate).

**Strains**	**Culture transfer**	**Cys input (mM)**	**Biomass_max_ (mg L^–1^)**	**Acetate titer (mM)**	**Productivity_max_ (mM day^–1^)**	**Yield_per_ biomass_max_ (mg L^–1^/ mg L^–1^)**
100%	–	3.2	95.7 ± 4.7	4.03 ± 0.11	1.38 ± 0.14	2.49 ± 0.18
50%	3	3.2	71.5 ± 7.1	7.14 ± 0.33	1.32 ± 0.07	5.94 ± 0.64
25%	3	3.2	43.7 ± 1.5	5.34 ± 0.57	1.46 ± 0.13	6.95 ± 0.99
100%	–	1.9	104.7 ± 5.6	3.91 ± 0.22	0.68 ± 0.04	3.07 ± 0.16
50%	17	1.9	68.8 ± 1.5	5.77 ± 0.28	0.81 ± 0.10	4.95 ± 0.23
25%	17	1.9	58.9 ± 5.6	12.59 ± 0.22	1.42 ± 0.08	12.64 ± 0.70

*The data represent the average of triplicate cultures and the standard deviation. See Supplementary Table S2 in the Supplementary Material for statistical analysis of relevant comparisons.*

After 17 culture transfers, the 25% strain clearly improved its performances in acetate production and cell growth. The final titer was 2.4–fold higher than the one obtained after only three transfers (*P*-value < 0.0001), while the yield per maximum biomass was approximately doubled (*P*-value = 0.0012). The maximum productivity stayed similar (difference not statistically significant), but a high production rate (in range of 1–1.4 mM day^–1^) was maintained for the entire cultivation period (Figure 4D). Furthermore, after 8 days of cultivation, small quantities of butyrate were produced, reaching a final titer of 0.43 ± 0.02 mM. Butyrate production started at acetate concentrations higher than 8 mM, in accordance with the previous work of Cypionka and Pfenning (Cypionka and Pfennig, 1986). The 50% strain, on the contrary, exhibited decreased performance compared to the 3rd transfer characterization (Table 2).

### Investigation of *D. orientis* Bioelectrosynthetic Acetate Production

We evaluated the performances of both 50 and 25% sulfate evolved strains at E_cath_ of −900 mV potentiostatic control and with a stable cathodic current at −15 mA of galvanostatic control. The goal of these experiments was to understand if acetate production in BES only comes from cysteine fermentation or if the adapted *D. orientis* strains are able to perform bioelectrosynthesis using CO_2_ and cathodically generated H_2_. Following Eq. S4, clear bioelectrosynthesis can be stated if the acetate titers were higher than the cysteine consumed.

**FIGURE 5:**
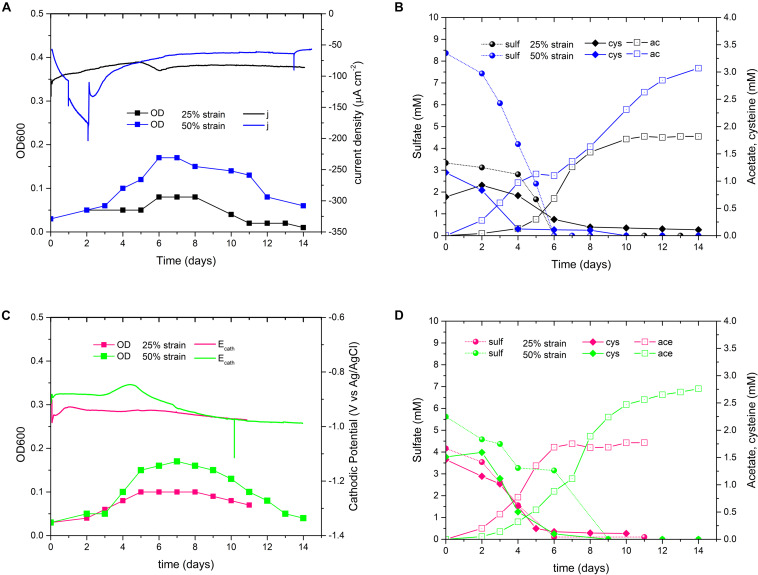
Comparison of *D. orientis* adapted strains biocathodes with potentiostatic and galvanostatic control. –900 mV Biocathodes inoculated with the 7th culture transfer of 50% sulfate strain and with the 8th culture transfer of the 25% sulfate strain: **(A)** OD and current density trends; **(B)** sulfate reduction, cysteine consumption and acetate production profiles. The best-performing reactors of the 50 and 25% strains are shown. See Supplementary Figure S5 for all the replicates. –15 mA Biocathodes: **(C)** OD and cathodic potential profiles and **(D)** sulfate reduction, cysteine consumption and acetate production profiles of 50% strains (13th culture transfer as inoculum) and 25% strains (13th culture transfer as inoculum). See Supplementary Figure S6 for replicate reactors.

The tests were performed in triplicate with the 7th 50% and the 8th 25% culture transfer, respectively, but the replicates behaved very differently in term of current consumption, biomass growth and acetate production (the best replicates are shown in Figures 5A,B, the others in Supplementary Figure S5). If we consider the best replicates (Figure 5B), the 50% biocathode outperformed the 25% for the final acetate titer (not statistically significant considering all replicates), while the latter exhibited a higher acetate yield per biomass (not statistically significant considering all replicates, see Table 3 and Supplementary Table S3). The maximum acetate productivity values of both strains were similar to the wild type biocathodes, and always occurred before cysteine depletion (Figure 5B). Yet, after complete cysteine and sulfate consumption, the 50% biocathodes exhibited a second acetate production phase lasting until the end of the test. The final acetate titer of the best performing 25% biocathode exceeded the cysteine input by ∼200%, while the one of the best performing 50% biocathode by ∼165%. Thus, both reactors clearly showed electrosynthetic acetate production, and similar values of cathodic current density [j_average_(50%) = −100 μA cm^–2^, j_average_(25%) = −86 μAcm^–2^].

**TABLE 3:** Performances of biocathodes inoculated with *D. orientis* strains adapted to sulfate-limiting growth conditions.

**Experiment**	**Δcys (mM)**	**Biomass_max_ (mg L^–1^)**	**Titer_acet_ (mM)**	**Max productivity_acet_ (mM day^–1^)**	**Acetate yield per biomass_max_ (mg L^–1^/ mg L^–1^)**	**CE_acet_ (%)**
−0.9 v 100% strain	0.90 ± 0.20	47.27 ± 4.66	1.01 ± 0.41	0.34 ± 0.08	1.28 ± 0.57	3.21 ± 0.57
−0.9 v 7th 50% strain	1.12 ± 0.03	25.73 ± 13.46	1.72 ± 1.32	0.35 ± 0.07 0.19 ± 0.17	3.49 ± 1.30	5.32 ± 0.83
−0.9 v 8th 25% strain	0.73 ± 0.03	13.17 ± 5.60	1.05 ± 0.67	0.34 ± 0.21	5.18 ± 2.77	2.73 ± 1.29
−15 ma 13th 50% strain	2.01	37.85	2.74	0.44 0.64	4.28	4.45
−15 ma 13th 25% strain	1.45	17.65	1.59	0.49	5.36	3.38
−15 ma 16th 50% strain +na-HCO_3_	1.13	27.08	2.94	0.68 0.64	6.52	5.28
−15 ma 15th 25% strain +na-HCO_3_	1.41	14.96	2.7	1.22	11.49	5.71

*The data represent the average of duplicate or triplicate reactors and where possible the standard deviation. See Supplementary Table S3 in the Supplementary Material for statistical analysis of relevant comparisons.*

Consequently, we decided to switch to a fixed cathodic current of −15 mA (=96 μA cm^–2^). The biocathodes were inoculated with the 13th culture transfer of both adapted strains (Figures 5C,D). Both biocathodes were more reproducible, especially regarding growth and acetate production profile (replicates Supplementary Figures S6A,B). The 50% biocathodes outperformed the 25% for the final acetate titer and for biomass production (Table 3), while the latter showed a higher acetate yield per biomass. In both adapted strains, the maximum productivity increased in comparison to the potentiostatically controlled biocathodes (Table 3), and the 50% biocathodes exhibited a maximum value of 0.64 mM day^–1^ during the second production phase with a final electrosynthetic acetate titer of on average 0.74 mM.

**FIGURE 6:**
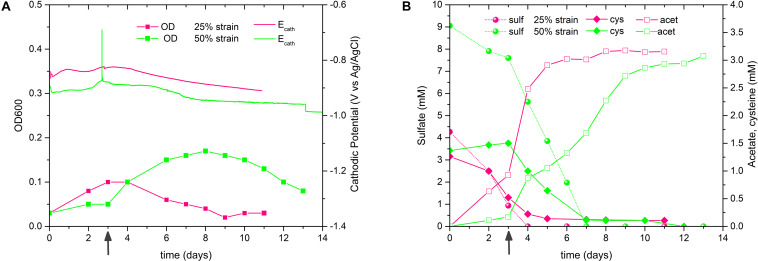
Performance of *D. orientis* adapted strains biocathodes with pulsed Na-bicarbonate and –15 mA applied cathodic current. **(A)** OD and cathodic potential profiles and **(B)** sulfate reduction, cysteine consumption and acetate production profiles. 50% sulfate biocathodes were inoculated with the 16th culture transfer, while 25% sulfate biocathodes with the 15th culture transfer. Dark gray arrows indicate the second pulse of 1 g L^– 1^ Na-bicarbonate. See Supplementary Figure S6 for replicate reactors.

Next, we increased the availability of CO_2_ in the reactors by doubling the normal concentration of Na-bicarbonate in the catholyte of 1 gL^–1^ with a second pulse after 3 days of experiment. It should be noted that the catholyte should be always CO_2_ saturated thanks to the continuous flushing of CO_2_/N_2_ (20:80) at flow rate of 50 mL min^–1^. The experiment was inoculated with the 16th and 15th culture transfers of 50 and 25% strains, respectively, and −15 mA cathodic current was applied. As shown in Figure 6, Supplementary Figures S6C,D, and Table 3, all these biocathodes exhibited a greater electrosynthetic acetate production than the previous BES tests (average duplicate: 1.81 mM the 50% strain and 1.29 mM the 25% strain). Furthermore, the 25% biocathodes drastically increased the maximum acetate productivity and the acetate yield per biomass, reaching values very similar to non-electrochemical serum bottles experiments (Tables 2 and 3): 1.22 mM day^–1^ and 11.49 mg L^–1^/mg L^–1^, respectively.

**FIGURE 7:**
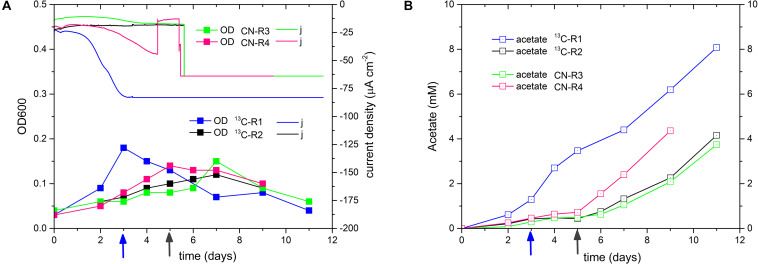
Performance of ^13^C labeling BES experiments, inoculated with the 18th culture transfer of 25% sulfate *D. orientis* strain. **(A)** current density and OD trends; **(B)** acetate production profile. ^13^C-R are biocathodes with Na-H^13^CO_3_, while CN-R are controls with normal Na-H^12^CO_3_. The second pulse of bicarbonate was added at day 3 in ^13^C-R1 (blue arrow), and at day 5 in the other reactors (gray arrow).

A final confirmation of *D. orientis’* bioelectrosynthesis ability was obtained through ^13^C-labeling BES experiments with the 18th culture transfer of the 25% strain. CO_2_ is the substrate of the Wood-Ljungdahl pathway (Ragsdale and Pierce, 2009; Berg, 2011). Dissolved CO_2_ can enter in *D. orientis* cells by membrane diffusion, while bicarbonate enters via an ABC-type transporter. As for other acetogens, *D. orientis* can convert intracellular bicarbonate to CO_2_ via carbonic anhydrases (Schnappauf et al., 1997; Pander et al., 2019). Since continuous flushing of ^13^CO_2_ gas would be prohibitively expensive, Na-H^13^CO_3_ was used and the flow rate of CO_2_/N_2_ gas feeding was decreased to 15 mL min^–1^. To avoid basification problems, 0.1 M MOPS buffer was added in the catholyte and the biocathode start-up was performed at E_cath_ −900 mV (Figure 7). Two reactors with Na-H^12^CO_3_ were run as controls. A 2nd pulse of Na-HCO_3_ was added during *D. orientis* middle/late exponential growth phase. Afterward, BES control was switched to a fixed applied current of −10 mA (−13 mA for ^13^C-Reactor 1, since it exhibited a much higher current during potentiostatic control).

The new BES experimental set-up resulted in a drastic increase of the maximum biomass generated (−13 mA biocathode = 49.97 mg L^–1^ and −10 mA biocathodes_average_ = 38.30 ± 4.11 mgL^–1^) that yielded the highest final acetate titers of all the tests reported in this study (−13 mA biocathode = 8.08 mM and −10 mA biocathodes_average_ = 4.09 ± 0.32 mM) (see Supplementary Table S3 for statistical analysis). Maximum productivities were 0.92 ± 0.08 mM day^–1^ at the end of the test for the −10 mA biocathodes and 1.41 mM day^–1^ for the −13 mA biocathodes right after the bicarbonate pulse. The acetate yield per biomass_max_ was lower (−13 mA biocathode: 9.55 mg L^–1^/ mg L^–1^ and −10 mA biocathodes: 6.38 ± 1.01 mgL^–1^/ mgL^–1^) in comparison to the previous BES test with the 25% strain 15th culture transfer (Table 3).

The atom percent ^13^C of derivatized acetate samples was measured by GC-MS/MS. Isotope ratio analysis was performed using the fragment ions CH_3_CO^+^ and CH_3_COOH, having mass-to-charge (m/z) ratios of 43 (M_0_), 44 (M_0__+__1_) and 61 (M_0_), 62 (M_0__+__1_), respectively. Supplementary Table S4 reports the atom percent ^13^C of the acetate produced by *D. orientis* biocathodes and in 2.5 mM and 5 mM acetate standards. Control reactors with only ^12^C-bicarbonate showed a ^13^C% similar to acetate standards and corresponding, thus, to ^13^C natural abundance. The samples taken after the addition of the 2nd pulse of bicarbonate in ^13^C- biocathodes exhibited an atom percent ^13^C 2-folds higher than ^12^ C- biocathodes (difference statistically significant, see Supplementary Tables S4, S5), confirming that some of the acetate was formed with the provided Na-H^13^CO_3_. These results are in accordance with the fact that, in our experimental conditions, *D. orientis* cells receive a dominant carbon supply directly from ^12^CO_2_ gas feeding compared to ^13^CO_2_ provided from ^13^C-bicarbonate. Estimate calculations on the availability of ^13^CO_2_ and ^12^CO_2_ in the catholyte based on the chemical equilibria are reported in the Supplementary Material Section 3.

## Discussion

The data presented in this work demonstrated the ability of *D. orientis* to perform a mediated electron uptake from cathodes in the presence of abiotically produced or biologically induced H_2_. High planktonic biomass production and high sulfate reduction were just observed in biocathodes poised more negative than −850 mV. In these conditions, H_2_ was even detected in the cathodic headspace, and, thus, it most likely represents the electron donor for *D. orientis* metabolism. Moreover, no biofilm, typically required for direct electron uptake, was observed on the graphite electrode surface during SEM imaging.

This conclusion was confirmed by metallic iron corrosion experiments, which can help in the understandings of microbial EEU mechanisms: if a direct electron uptake pathway is present, microbes are able to use Fe^0^ as sole electron donor. Very recently, Tang et al. (2019) has demonstrated direct metal electron uptake via c-OMCs in the autotrophic strain *Geobacter sulfurreducens* ACL. Evidences of similar EEU mechanism have been reported for two iron-corroding SRB, *Desulfopila corrodens* strain IS4 (Beese-Vasbender et al., 2015) and *Desulfovibrio ferrophilus* IS5 (Deng and Okamoto, 2018; Deng et al., 2018).

*Desulfosporosinus orientis* was not able to use Fe^0^ as extracellular electron donor, which is supported by *D. orientis’* belonging to the cytochrome-poor group of SRB, lacking c-OMCs and periplasmatic multihemes cytochromes (Rabus et al., 2015; Agostino and Rosenbaum, 2018). The electron transport chain is rather based on membrane-bound hydrogenases and formate dehydrogenases associated to the inner membrane through a *b*-type cytochrome that directly reduces the menaquinone pool (Rabus et al., 2015; Agostino and Rosenbaum, 2018). Specifically, the genes Desor_0303, Desor_0911, and Desor_4119 code for the membrane-associated [NiFe]-hydrogenases of group 1, responsible of hydrogen uptake (Peters et al., 2015). Moreover, a [NiFe]-hydrogenase of group 4f (Desor_1267), with a predicted activity related to H_2_-evolution, is also present in its genome. This hydrogenase is a component of the anaerobic formate hydrogen lyase complex (Desor_1265 to Desor_1270) that couples oxidation of formate to the reduction of protons to H_2_ (Sinha et al., 2015). After microbial cell lysis, the release of this complex in the cathodic environment, as well as of the cytoplasmatic [FeFe]-electron bifurcating hydrogenases (Desor_1568, Desor_4949, Desor_5363) could facilitate biotic, non-microbial H_2_ production by decreasing the hydrogen evolution overpotential (Deutzmann et al., 2015). This possible EEU mechanism in *D. orientis* should be further investigated via BES experiments with cell-free spent medium as catholyte (Rowe et al., 2019). Moreover, very recently, the maintenance of low H_2_ partial pressures via microbial H_2_ consumption was proposed as additional mechanism by which hydrogenotrophic microorganisms could increase the H_2_ evolution rate on a cathode or Fe^0^ (Philips, 2020).

The second goal of this study was to characterize the ability of *D. orientis* for bioelectrochemical sulfate reduction and acetate production with CO_2_ as carbon source.

All previous works related to bioelectrochemical sulfate removal were carried out using undefined mixed-community autotrophic biocathodes, as we recently reviewed (Agostino and Rosenbaum, 2018). The biocathodic inoculum usually consisted of consortia enriched from wastewater treatment sludge, sewer and sediment, mostly dominated by *Desulfovibrio* species. In BES research, mixed communities often outperform pure culture biocatalysts, such as for electricity generation in Microbial Fuel Cell and for acetate production in MES. Nevertheless, in this case, *D. orientis* maximum sulfate reduction rate (SRR) (0.19 ± 0.04 g L^–1^ day^–1^ at E_cath_ −900 mV) was very similar to mixed-community biocathodes (Agostino and Rosenbaum, 2018), except for the works of the Freguia group who obtained a SRR_max_ of 5.6 g L^–1^ day^–1^ but at very negative E_cath_ −1300 mV (Pozo et al., 2017).

Regarding acetate production with pure cultures, the acetogenic strains *Sporomusa ovata* and *C. ljungdahlii* are producing the highest amount of acetate, with maximum production rates of 0.88 and 2.4 mM day^–1^, at cathodic potentials negative enough to permit abiotic H_2_ evolution (Bajracharya et al., 2015; Aryal et al., 2017). In addition, acetate productivity by *Sporomusa*-driven MES can further increase to 3.12 mM day^–1^, after its adaptation to methanol as only carbon and energy source (Tremblay et al., 2015).

In optimal sulfate conditions, *D. orientis* seems to generate acetate only via cysteine fermentation, with a maximum productivity of 0.34 mM day^–1^. However, after successful ALE in sulfate-limiting conditions, both 25 and 50% adapted *D. orientis* strains (15th and 16th culture transfer) have shown electrosynthetic acetate production biocathodes with fixed current flow of −15 mA and 2 g L^–1^ of Na-HCO_3_. The maximum acetate productivity of the 50% sulfate biocathodes (0.64 mM day^–1^) was very similar to the values achieved by some *Sporomusa* species (*S. acidovorans and S. malonica*) (Aryal et al., 2017). The maximum productivity of the 25% sulfate *D. orientis* biocathodes was double (1.22 mM day^–1^) compared to 50% biocathodes.

We confirmed electrosynthetic acetate production from CO_2_ in a modified BES set-up with ^13^C-labeling experiments. In this test, continuous acetate production even into the *D. orientis* stationary/dying phase was obtained with the 25% biocathodes, resulting in the highest acetate final titers (−13 mA biocathode = 8.08 mM and −10 mA biocathodes_average_ = 4.09 mM) and productivities (−13 mA biocathode = 1.41 mM day^–1^ and −10 mA biocathodes_average_ = 0.92 ± 0.08 mM day^–1^) of all the tests reported in this work. Since this performance is similar to other acetogenic pure-cultures, *D. orientis* might compete well with *S. ovata* or *C. ljungdahlii* for MES applications. The main differences in this modified BES set-up, compared to the other experiments of our study, consisted of the addition of MOPS buffer and the utilization of a lower CO_2_ feeding flow rate. Independently of the presence or not of MOPS buffer, the catholyte pH always rose to values of 7.4–7.5 along the experiments and, thus, extracellular pH cannot possibly be the limiting factor. Acetate production performances seem more correlated to the CO_2_ flow rate and the amount of H_2_ abiotically produced or biologically induced during the biocathodic start-up phase. The stoichiometry ratio of H_2_ and CO_2_ for acetate production is 4 to 2. High amount of dissolved CO_2_ and /or H_2_ could inhibit *D. orientis* growth and production performances, as demonstrated in high pressure gas fermentation studies with other acetogenic strains (Weuster-botz, 2016; Oswald et al., 2018). Further investigations using CO_2_ and H_2_ sensors inside the catholyte could clarified, which of these two elements represents the limiting and/or inhibiting factor for acetate production within our *D. orientis* biocathodes set-up.

During ALE experiments, the 25% adapted strain has shown butyrate production ability in serum bottles with H_2_ and CO_2_ atmosphere. Butyrate is a C4 carboxylic acid with higher market value than acetate. It has multiple applications in pharmaceutical, chemical, food and cosmetic industries (Dwidar et al., 2012). Moreover, butyrate is becoming a valuable feed supplement for animal production, as alternative of in-feed antibiotics (Bedford and Gong, 2018). In chemical industry, its major application is in the production of cellulose acetate butyrate (CAB) polymers. Bioelectrosynthesis of butyrate from CO_2_-rich waste gas streams represents a recent and promising alternative to the current fossil-fuel-based commercial manufacturing (Ganigué et al., 2015; Jourdin et al., 2019). Consequently, future works will focus the attention on a better control of BES process parameters (e.g., CO_2_ feeding strategy, controlled electrochemical H_2_ dosing, stirring rate and high cells density inoculum) in order to achieve and optimize electrosynthetically butyrate production with *D. orientis* biocathodes.

In deep electrophysiological characterization of novel pure culture electroautotrophs are still pretty scarce. Growing pure culture autotrophic microbes in BES is a hard task. In our opinion, this study provides a valuable contribute to the field of electromicrobiology. The more we know, and we learn on the physiology and the metabolic features of these fascinating bacteria, the more we can accelerate the development of BES-based technologies for sustainable bioproduction from CO_2_-rich waste streams.

## Data Availability Statement

All relevant data are included in this manuscript and the corresponding Supplementary Material.

## Author Contributions

VA coordinated the study, designed, conducted and analyzed the experiments, and prepared the draft of the manuscript. AL conducted and analyzed the part of the experiments. BB conducted the cysteine quantification analyses and revised the manuscript. AP supervised and optimized the GC-MS/MS analyses. VR conducted the SEM sample preparation and analyses. LB discussed the work and revised the manuscript. MR conceived the work, advised on the experimental plan, discussed the experiments, revised the manuscript, and provided the funding.

## Conflict of Interest

The authors declare that the research was conducted in the absence of any commercial or financial relationships that could be construed as a potential conflict of interest.
