# Supplementary material for: Electrophysiology of the Facultative Autotrophic Bacterium Desulfosporosinus orientis
Source: Front Bioeng Biotechnol. 2020 May 19;8:457. doi: 10.3389/fbioe.2020.00457 (PMC7248197; doi:10.3389/fbioe.2020.00457)
Supplement: Supplementary file 1 [file Data_Sheet_1.docx]

Supplementary Material

**Electrophysiology of the facultative autotrophic bacterium *Desulfosporosinus orientis***

Agostino Valeria^1,^ ^#^ , Lenic Annika^1^, Bardl Bettina^2^, Rizzotto Valentina^3^, Phan An N. T.^1^, Blank Lars M.^1^, Rosenbaum Miriam A.^2, 4^ ^*^

^1^ Institute of Applied Microbiology iAMB, Aachen Biology and Biotechnology, RWTH Aachen University, Aachen, Germany

^2^ BioPilot Plant, Leibniz Institute for Natural Product Research and Infection Biology – Hans Knöll Institute, Jena, Germany

^3^ Institute of Inorganic Chemistry, RWTH Aachen University Aachen, Germany

^4^ Faculty of Biological Sciences, Friedrich Schiller University, Jena, Germany

*Correspondence:

Miriam A. Rosenbaum

[miriam.rosenbaum@leibniz-hki.de](mailto:miriam.rosenbaum@leibniz-hki.de)

^#^ Present address: Valeria Agostino, Centre for Sustainable Future Technologies, Fondazione Istituto Italiano di Tecnologia, Torino, Italy

**Supplementary data, tables and figures**

This supplementary material contains supplemental data, tables and figures. Specifically, in Additional Data, details on the results related to corrosion experiment (Section 1), cysteine influence on biocathodic acetate production (Section 2) and ^13^C labelling BES experiment (Section 3) are reported. Supplementary Tables contain statistical analysis of relevant and possible (triplicate) data comparison (Table S1-3 and S5) and the atom percent ^13^C of the acetate produced by *D. orientis* biocathodes (Table S4). Supplementary Figures show the abiotic H_2_ productivity in our H-type BES reactors and a picture of the prototype used (Fig. S1), the replicates of BES reactors experiment reported in this work and SEM imaging of graphite electrodes (Fig. S2 and S4-S6), and the results of serum bottles corrosion experiment (Fig. S3).

**Additional data**

**Section 1. Corrosion experiment**

In microbial corrosion experiments, optical density cannot be used as growth monitoring parameter, as Fe^0^ reacts easily with sulfide and produces FeS black precipitates. Consequently, *D. orientis’* sulfate reduction to sulfide and acetogenesis were evaluated.

Even if Fe^0^ was the only electron donor present in the cultures, H_2_ can be abiotically produced from anaerobic corrosion of Fe^0^ in water at neutral conditions (Enning et al. 2012; Dinh et al., 2004):

Fe^0^ 🡪 Fe^2+^ +2e^-^ (S1)

2H_2_O +2e^-^ 🡪 H_2_ + 2OH^-^  (S2)

Moreover, if sulfide is present in the medium, acceleration of abiotic corrosion and H_2_ production occurs, as hydrogen sulfide is a highly oxidative compound (Enning et al., 2012):

H_2_S+Fe^0^ 🡪 FeS +H_2_ (S3)

Consequently, the H_2_ in the headspace of *D. orientis* cultures and abiotic controls (with and without the addition of Na-sulfide (18 mM) was monitored.

Figure S3 reports the sulfide, acetate and H_2_ formation profiles, expressed in mmoles of electron equivalent, considering the headspace volume for H_2_ and the liquid volume for sulfide and acetate. H_2_ was detected in both abiotic controls and it increased over the time. As expected, the addition of Na-sulfide in the medium allowed a 2.6-fold acceleration of abiotic chemical H_2_ production, with a maximum production rate of 190 mmol eeq day^-1^, compared to 72 mmol eeq day^-1^in the Na-sulfide-free abiotic controls (Figure S3 B). No H_2_ was detected in *D. orientis* Fe^0^ cultures, indicating its consumption.

The maximum rate of sulfide formation in *D. orientis* Fe^0^ cultures (195 mmol eeq day^-1^) was very similar to the abiotic H_2_ production rate of Na-sulfide controls (Figure S3 A). Sulfide was generated also in the biotic controls with N_2_/CO_2_ atmosphere and no Fe^0^, but with a lower maximum productivity (120 mmol eeq day^-1^). In both biotic conditions, the sulfide production rate decreased drastically after 3 days of cultivation. Acetate production was not detected in these experiments. In contrast clear sulfide (measured via sulfate reduction) and acetate production was detected in a positive biotic control with a H_2_/CO_2_ atmosphere and no iron (Figure S3 C). There is likely no growth with Fe^0^ as electron donor. Biological activity in F^0^ cultures can be explained solely by abiotic hydrogen provision.

SEM analysis showed no microbial colonization of the F^0^ granules surfaces, giving a further confirmation of the inability of *D. orientis* in using Fe^0^ as extracellular electron donor (Figure S3 D-F). The Fe^0^ surface was covered by precipitates, most probably FeS. Similar lamellas were observed also in the abiotic controls with and without Na-sulfide (Figures S3 F and E). The abiotic control medium never turned to blackish color but a whitish precipitation was observed, suggesting the presence of FeCO_3_ of Fe(OH)_3_ precipitates (De Paepe et al., 2018; Georg et al., 2018).

**Section 2. Cysteine influence on biocathodic acetate production**

We evaluated biocathodic acetate production in relation to cysteine consumption, testing two different cysteine input concentrations in the reactor medium (3.2 mM and 1.9 mM). *D. orientis* biocathodes completely consumed the cysteine in the medium, independently of its starting concentration and the E_cath_ applied, as reported in Figure 3. The final titer of acetate was dependent on the input cysteine concentration, as well as on the E_cath._ In addition, the final acetate titer was never higher than the input cysteine concentration (Table 1). The -900 mV biocathodes with 3.2 mM of cysteine exhibited the highest acetate titer of 2.5 mM after 12 days (Figure 3A).

A simple carbon and electron balance of acetate production from cysteine (without considering biomass formation) shows that 1 mole of acetate can be formed per mole of cysteine consumed:

C_3_H_7_NO_2_S + 3H_2_O🡪H_2_S + NH_4_^+^ + C_2_H_3_O_2_^-^+ HCO_3_^-^+ 3H^+^ + 2e^-^  (S4)

Sulfate removal, on the contrary, is independent from cysteine concentration input but only dependent on E_cath_ (Table 1). As shown in Figures 1B, 2B and S4D, -900 mV biocathodes with 3.2 mM cysteine input reduced approximately 8 mM of sulfate, while -900 mV biocathodes with 1.9 mM cysteine input removed 12 mM±1.3 mM of sulfate. Biomass formation was more dependent on E_cath_ than on cysteine input, as shown in Table 1.

**Section 3.** **Estimate calculations on the availability of ^13^CO_2_ and ^12^CO_2_ in the catholyte.**

- ^13^CO_2_ concentration originating from provided bicarbonate via carbonic acid dissociation equilibrium:

**[^13^CO_2_] = [H^+^]x[HCO_3_^-^] / K_1_ = 0.81 mM**

with pH= 7.5, [HCO_3_^-^]= 12 mM, and K_1_= 4.71x10^-7^ mol/L (Saruhashi, 1995)

- Max ^12^CO_2_ solubility from gas purging via [van 't Hoff equation](https://en.wikipedia.org/wiki/Van_%27t_Hoff_equation) applies to Henry's law constant:

**[^12^CO_2_]_aq_ = K_Hc,p_ x P = 38.8 mM**

with K_Hc,p_ at 37°C = 0.038827437 M, and P=1 atm

Time to reach max ^12^CO_2_ solubility: V_cath_ / q = 2.44 hours

with V_catholyte_ = 440 mL, and q= 15 mL/min of 0.2 atm CO_2_.

- Expected maximum ^13^C%-acetate if all ^13^CO_2_ would have been utilized (assuming no flush-out of ^13^CO_2_):

**^13^C-acetate%_exp_= (([^13^CO_2_] / 2) x 100) / [acetate]_final_**

^13^C-acetate%_exp_ R1= 4.99%

^13^C-acetate%_exp_ R2= 10.39%

with [acetate]_final_ R1= 8.1 mM and R2= 4.2 mM

References:

Enning D, Venzlaff H, Garrelfs J, Dinh HT, Meyer V, Mayrhofer K, et al. Marine sulfate reducing bacteria cause serious corrosion of iron under electroconductive biogenic mineral crust. Environ Microbiol. 2012;14(7):1772–87.

Dinh HT, Kuever J, Mußmann M, Hassel AW, Stratmann M, Widdel F. Iron corrosion by novel anaerobic microorganisms. Nature. 2004;427(6977):829–32.

De Paepe K, Van den Driessche N, Rabaey K, Arends JBA, Philips J, Gralnick JA, et al. A novel *Shewanella* isolate enhances corrosion by using metallic iron as the electron donor with fumarate as the electron acceptor. Appl Environ Microbiol. 2018;84(20).

Georg S, Prévoteau A, Arends JBA, Philips J, Monballyu E, Rabaey K, et al. An *Acetobacterium* strain isolated with metallic iron as electron donor enhances iron corrosion by a similar mechanism as *Sporomusa sphaeroides*. FEMS Microbiol Ecol. 2018;95(2).

Saruhashi, K. Metabolism in natural waters, part2- Equilibrium, concentration ratio of carbonic acid substances dissolved in natural water. Meteorol. Geophys. Papers, 1955; (6):38-55.

**Supplementary Tables**

**Table S1** Statistical analysis (GraphPad Prism): two-tailed, unpaired, student's t tests (without assuming a consistent SD) were employed for comparison of means of the performances of -900 mV biocathodes and -800 mV biocathodes, in optimal sulfate concentration conditions and with 1.9 mM cysteine input. Number of t-tests: 5. In red, P value > 0.05.

|  | P value | | | | |
| --- | --- | --- | --- | --- | --- |
| Comparison | 10-days sulfate removal (%) | Max_sulfate_ red. rate  (mM day^-1^) | Tot. CE  (%) | j_max_  (µA cm^-2^) | Max. Biomass (mg L^-1^) |
| -900 mV vs -800 mV | 0.0002 | 0.0097 | 0.2167 | <0.0001 | 0.0002 |

**Table S2** Statistical analysis (GraphPad Prism): two-tailed, unpaired, student's t tests (without assuming a consistent SD) were employed for comparison of means of the performances of *D. orientis* strains adapted to sulfate-limiting growth conditions in serum bottles. Number of t-tests: 21. In red, P value > 0.05.

|  | P value | | |
| --- | --- | --- | --- |
| Comparison | Acetate titer  (mM) | Productivity_max_  (mM day^-1^) | Yield_per_ biomass_max_  (mg L^-1^/ mg L^-1^) |
| 100% vs 3^rd^50% strains | 0.0001 | 0.5430 | 0.0008 |
| 100% vs 3^rd^25% strains | 0.0174 | 0.5084 | 0.0015 |
| 3^rd^50% vs 3^rd^25% strains | 0.0091 | 0.1759 | 0.2120 |
| 100% vs 17^th^ 50% strains | 0.0008 | 0.1048 | 0.0003 |
| 100% vs 17^th^ 25% strains | <0.0001 | 0.0001 | <0.0001 |
| 17^th^ 50% vs 17^th^ 25% strains | <0.0001 | 0.0012 | <0.0001 |
| 3^rd^ vs 17^th^ 25% strains | <0.0001 | 0.6734 | 0.0012 |

**Table S3** Statistical analysis (GraphPad Prism): two-tailed, unpaired, student's t tests (without assuming a consistent SD) were employed for comparison of means of the performances of biocathodes inoculated with *D. orientis* strains adapted to sulfate-limiting growth conditions. Number of t-tests: 18. In red, P value > 0.05.

|  | P values | | |
| --- | --- | --- | --- |
| Comparison | Acetate titer  (mM) | Productivity_max_  (mM day^-1^) | Yield_per_ biomass_max_  (mg L^-1^/ mg L^-1^) |
| -0.9V 100% vs 50% 7^th^ CT | 0.4239 | 0.8785 | 0.0543 |
| -0.9V 100% vs 25% 8^th^ CT | 0.8756 | >0,9999 | 0.0753 |
| -0.9V 50%7^th^ CT vs 25% 8^th^ CT | 0.4295 | 0.9414 | 0.3929 |
| *MOPS 25% 18^th^ vs -0.9V 100% | 0.0005 | 0.0009 | 0.0016 |
| *MOPS 25% 18^th^ vs -0.9V 50% 7^th^ CT | 0.0391 | 0.0007 | 0.0384 |
| *MOPS 25% 18^th^ vs -0.9V25% 8^th^ CT | < 0.0001 | 0.0111 | 0.5197 |

**MOPS reactors: -0.9 V / -10 mA.*

**Table S4** Atom percent ^13^C of acetate derivatized samples from *D. orientis* biocathodes inoculated with the 18^th^ culture transfer of 25% strain. T= time in days

| **Na –Bicarbonate** | **Samples** | **Atom % ^13^C**  **(m/z 62-61)** | **Atom % ^13^C**  **(m/z 44-43)** |
| --- | --- | --- | --- |
| **-** | ^12^C-Acetate 2.5mM | 1.67 | 2.45 |
| **-** | ^12^C-Acetate 5mM | 1.71 | 2.62 |
| ^13^C_1 gl ^-1^ | R1_T0 | 1.89 | 2.23 |
| ^13^C_1 gl ^-1^ | R1_T3 | 1.98 | 2.36 |
| **^13^C_2 gl^-1^** | **R1_T5** | **4.36** | **4.91** |
| **^13^C_2 gl^-1^** | **R1_T7** | **3.69** | **4.31** |
| **^13^C_2 gl^-1^** | **R1_T11** | **3.14** | **3.57** |
| ^13^C_1 gl^-1^ | R2_0 | 1.55 | 2.28 |
| ^13^C_1 gl^-1^ | R2_T4 | 1.87 | 2.58 |
| **^13^C_2 gl^-1^** | **R2_T6** | **5.32** | **5.79** |
| **^13^C_2 gl^-1^** | **R2_T9** | **5.38** | **5.98** |
| **^13^C_2 gl^-1^** | **R2_T11** | **4.39** | **4.88** |
| ^12^C_1 gl^-1^ | CN R3_T0 | 1.72 | 2.27 |
| ^12^C_1 gl^-1^ | CN R3_T4 | 1.85 | 2.54 |
| ^12^C_2 gl^-1^ | CN R3_T6 | 1.96 | 2.40 |
| ^12^C_2 gl^-1^ | CN R3_T9 | 1.89 | 2.27 |
| ^12^C_2 gl^-1^ | CNR3_T11 | 1.77 | 2.37 |
| ^12^C_1 gl^-1^ | CN R4_T0 | 1.77 | 2.46 |
| ^12^C_1 gl^-1^ | CN R4_T4 | 1.71 | 2.34 |
| ^12^C_2 gl^-1^ | CN R4_T6 | 1.95 | 2.41 |
| ^12^C_2 gl^-1^ | CN R4_T9 | 1.38 | 2.40 |

**Table S5** Statistical analysis (GraphPad Prism): two-tailed, unpaired, student's t tests (without assuming a consistent SD) were employed for comparison of means of atom percent ^13^C of acetate derivatized samples from *D. orientis* biocathodes inoculated with the 18^th^ culture transfer of 25% strain. Number of t tests: 2.

|  | P value | Mean of ^13^C reactors* | Mean of ^12^C reactors* | Difference | SE of difference |
| --- | --- | --- | --- | --- | --- |
| m/z 62-61 | 0.0005 | 4.688 | 1.795 | 2.893 | 0.4296 |
| m/z 44-43 | 0.0003 | 5.248 | 2.37 | 2.878 | 0.3913 |

**^13^C reactors mean of samples R1T5, R1T7, R2T6, R2T9; ^12^C reactors mean of samples R3T6, R3T9, R4T6, R4T9.*

**Supplementary Figures**

**A** B


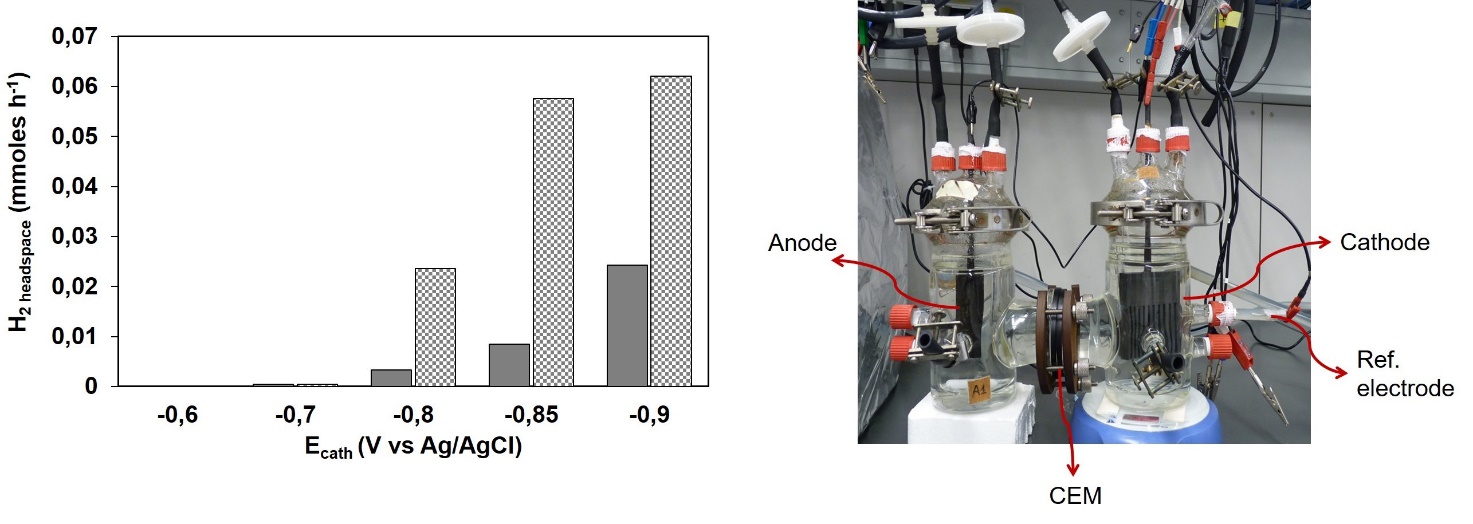


Figure S1 (A) Abiotic H_2_ productivity in H-type BES reactors at different cathodic polarization conditions. Each E_cath_ was maintained for 3 days and off-line headspace H_2_ quantification was performed before switching to the next E_cath_. The two replicates are shown in dark grey and checkered bars. (B) Image of H-type BES reactor used. CEM= cation exchange membrane, Reference electrode = Ag/AgCl (sat. KCl).


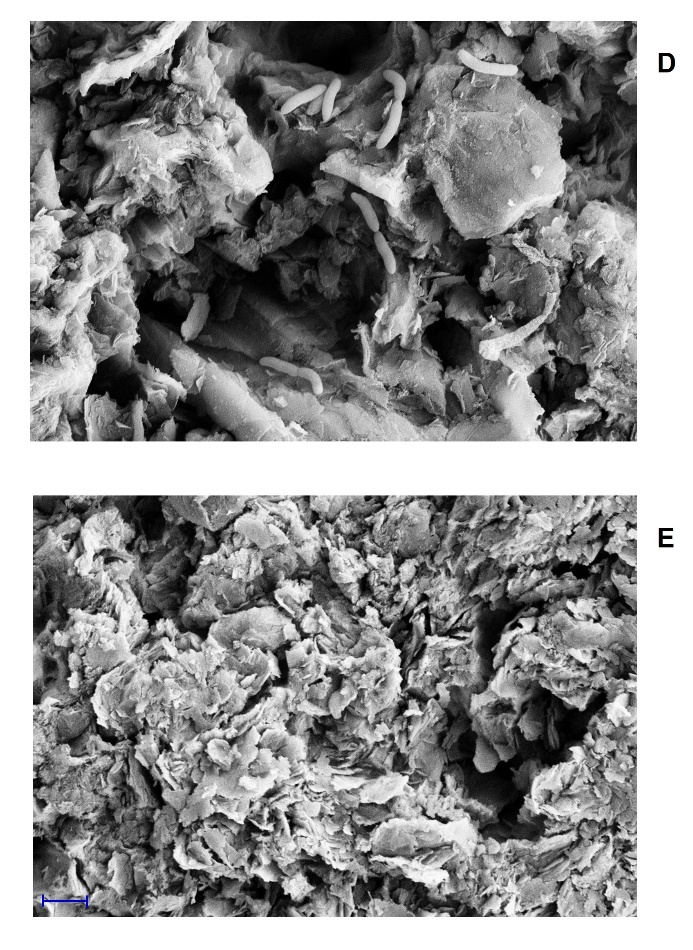

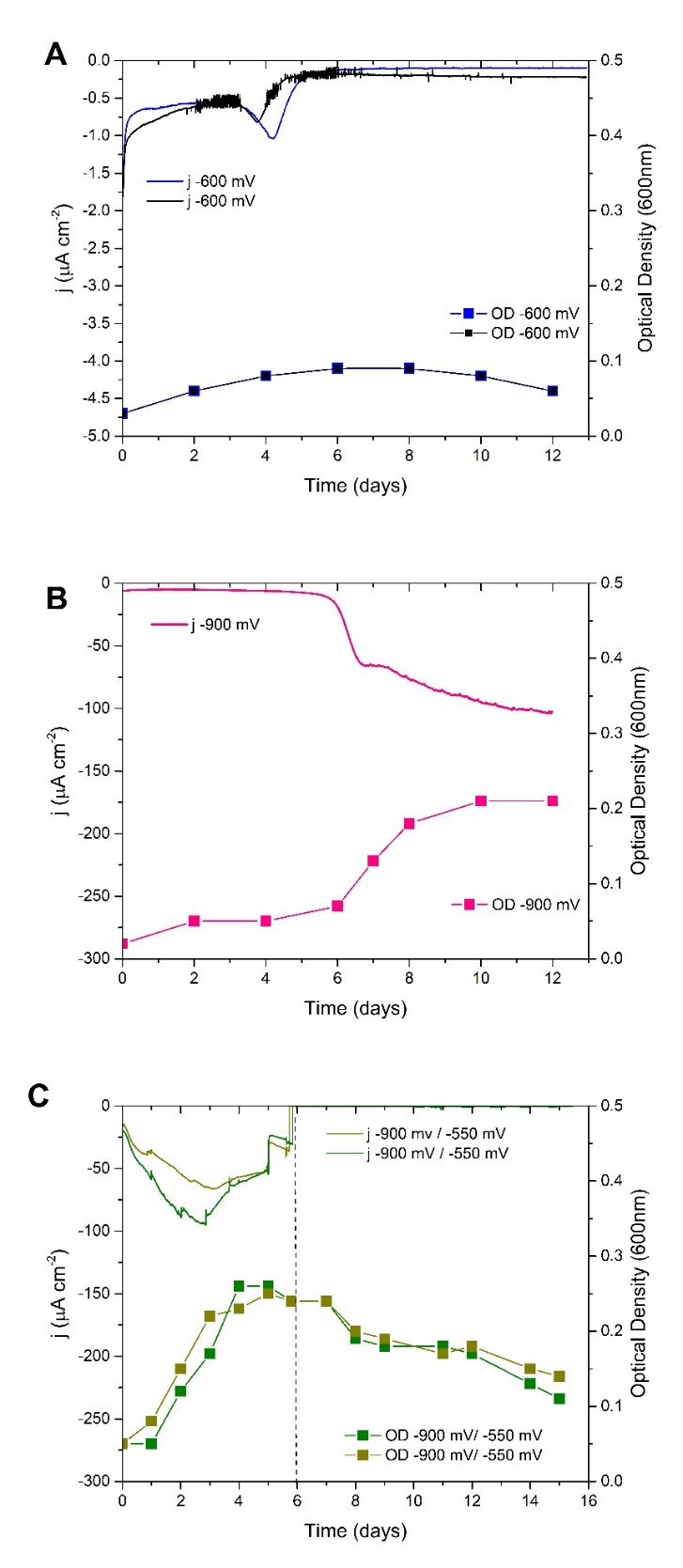
**Figure S2** Current density and OD trends of *D. orientis* biocathodes at different E_cath_, replicate reactors of Figure 1. **(A)** -600 mV biocathodes; **(B)** -900 mV biocathodes; **(C)** -900 mV/-550 mV biocathodes, the dashed line indicates the E_cath_ switch time point. SEM images of cathodic graphite electrodes at the end of experiments: **(D)** SEM images of *D. orientis* biocathodes at -900 mV and (**E)** -600 mV; Scale bars represent 2 µM, magnification =10 K X


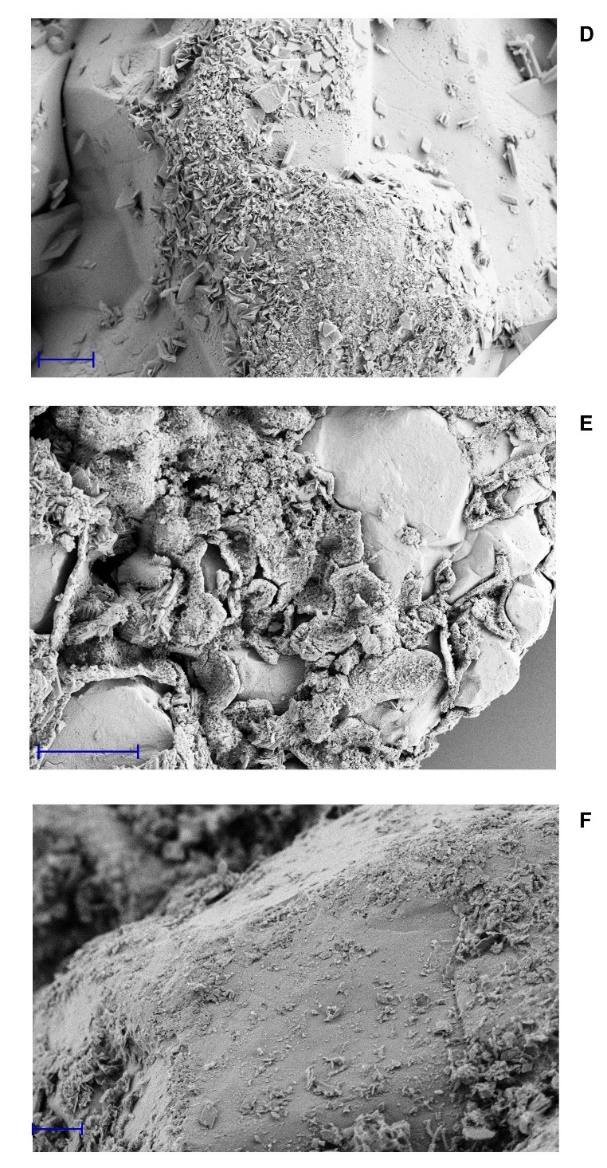

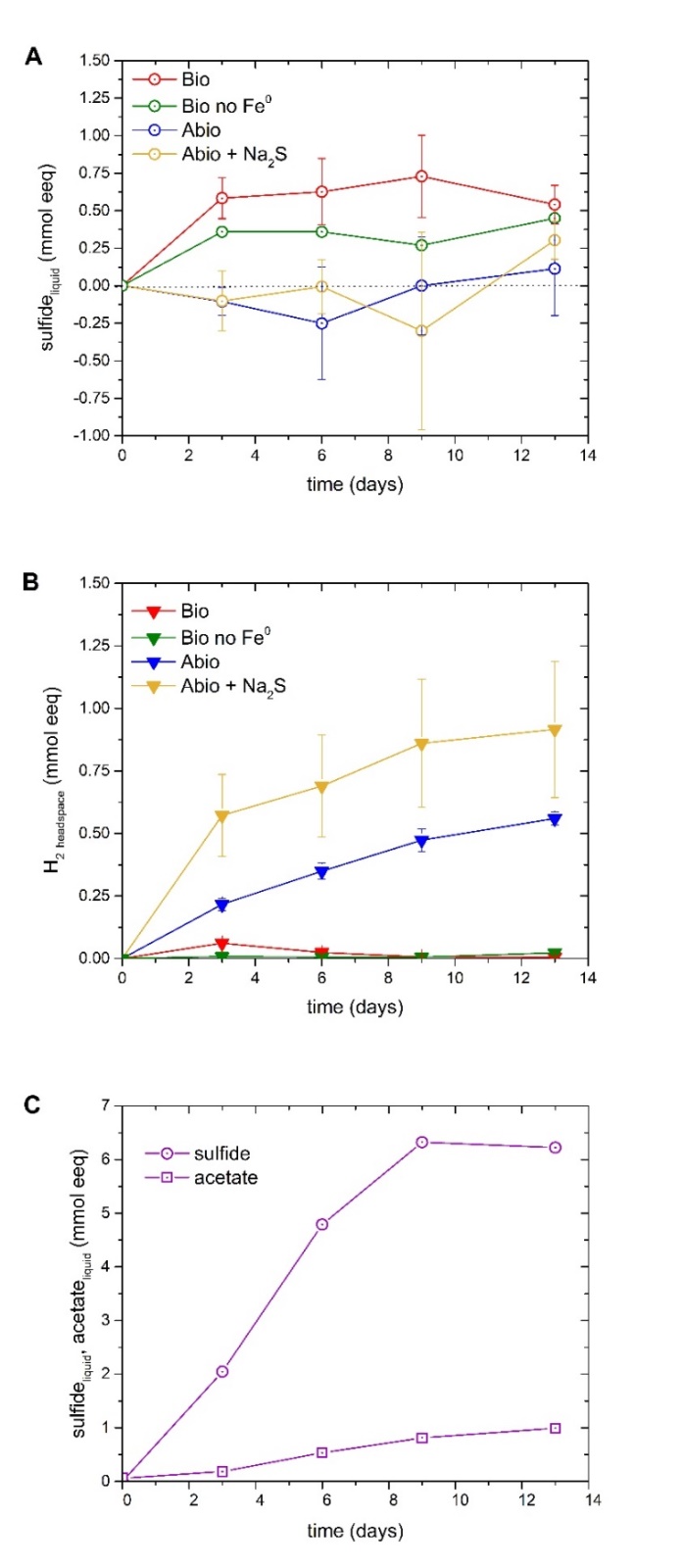


Figure S3 *D. orientis* corrosion experiments with 5g of Fe^0^ per 50 ml medium in N_2_/CO_2_ atmosphere against the following controls: two abiotic controls with and without the addition of 18 mM of Na-sulfide and a biotic control without Fe^0^. Quantification of (A) sulfide (via sulfate reduction) and (B) H_2_ formation profiles, expressed in mmoles of electron equivalent, considering the headspace volume for H_2_ and the liquid volume for sulfide and acetate; (C) *D. orientis* autotrophic culture control with H_2_/CO_2_ atmosphere. The data represent the average of triplicate or duplicate cultures and the error bars standard deviation. SEM images of iron granules at the end of experiments: (D) *D. orientis* autotrophic culture with N_2_/CO_2_ atmosphere; (E, F) abiotic controls with and without 18mM Na-Sulfide addition. Scale bars represent 10 µM (D and F) and 100 µM (E), magnification= 2.5 K X (D and F) and 500 X (E).


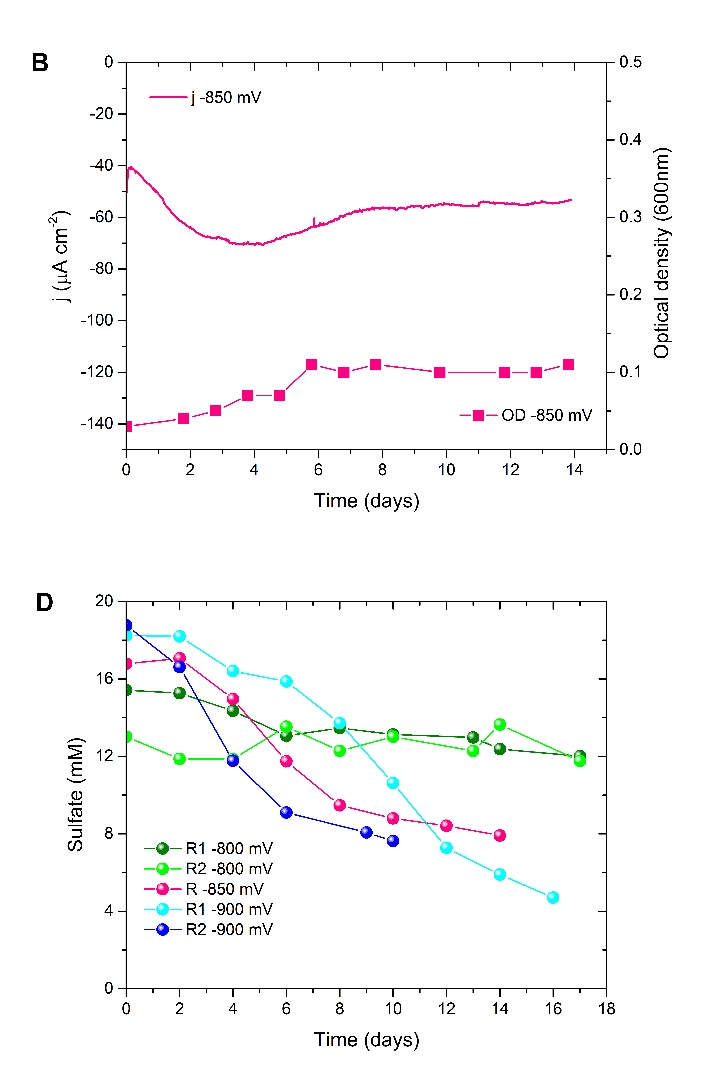

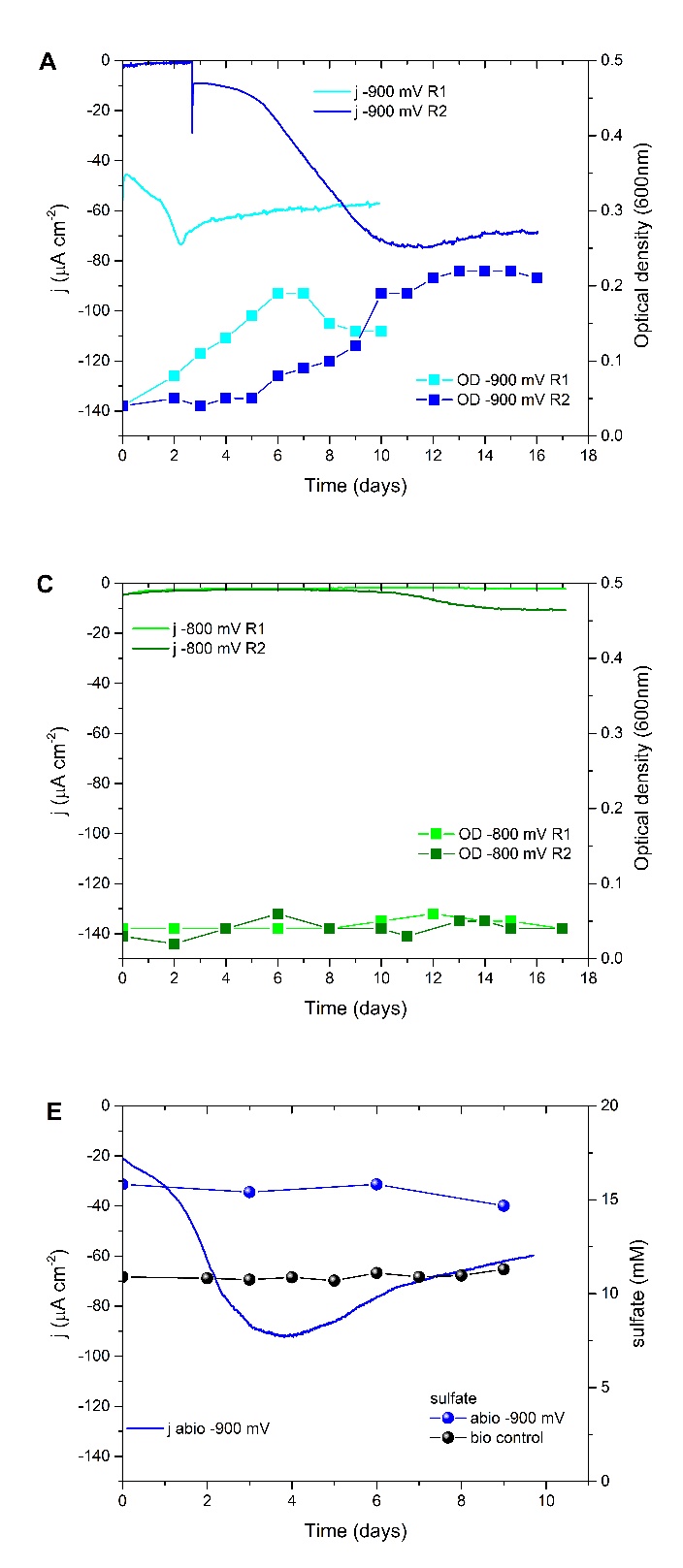
 **Figure S4** Replicates of *D. orientis* biocathodes at different E_cath_ with 1.9 mM cysteine input (Figure 2). (A) Current density and OD trends of -900 mV biocathodes; (B) -850 mV biocathodes; (C) -800 mV biocathodes; (D) sulfate reduction trend of *D. orientis* biocathodes; (E) current density and sulfate concentration profiles of abiotic -900 mV cathode and biotic control cathode without electrode.


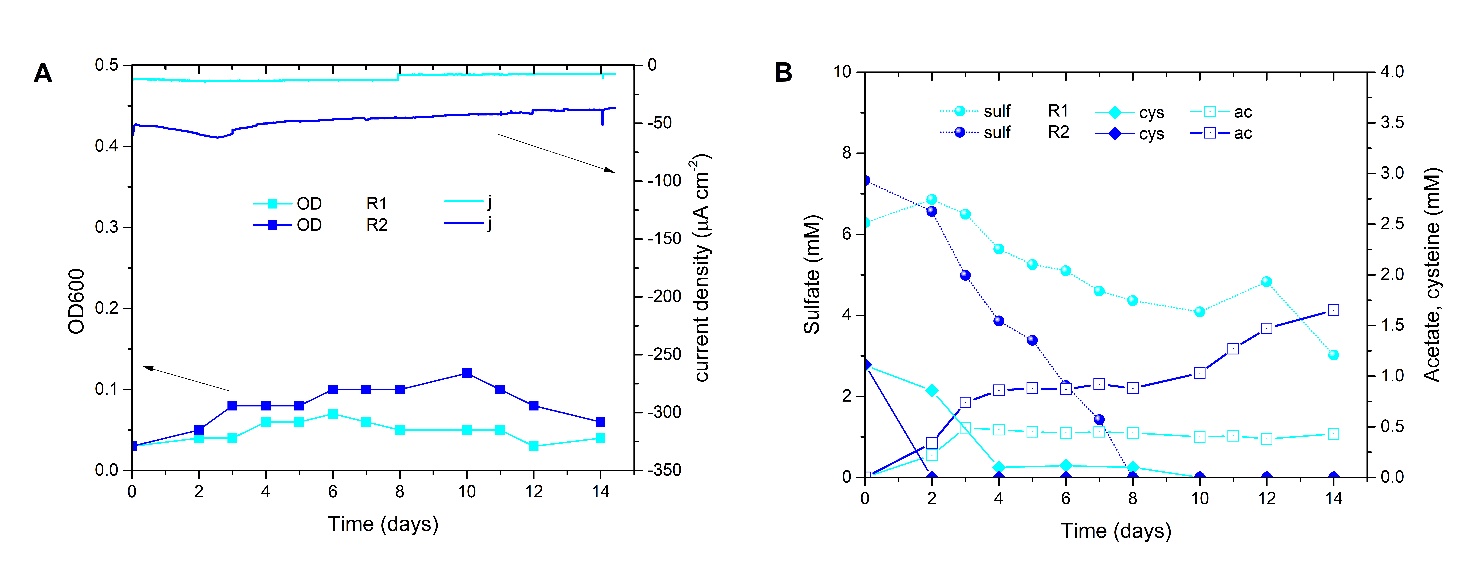

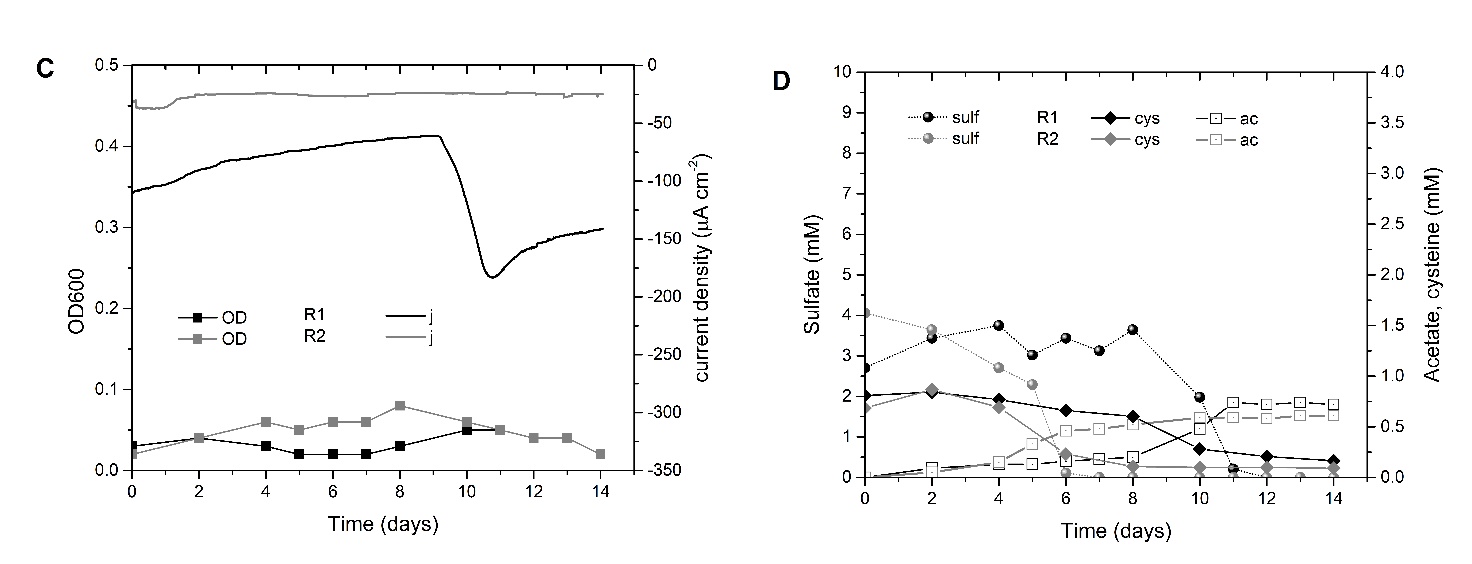


Figure S5 Replicates of -900 mV biocathodes inoculated with *D. orientis* adapted strains (Figure 5 A and B). 7^h^ culture transfer of the 50% strain as inoculum: (A) current density and growth profile; (B) sulfate, cysteine and acetate concentration in the catholyte. 8^th^ culture transfer of the 25% strain as inoculum: (C) current density and growth profile; (D) sulfate, cysteine and acetate concentration in the catholyte.


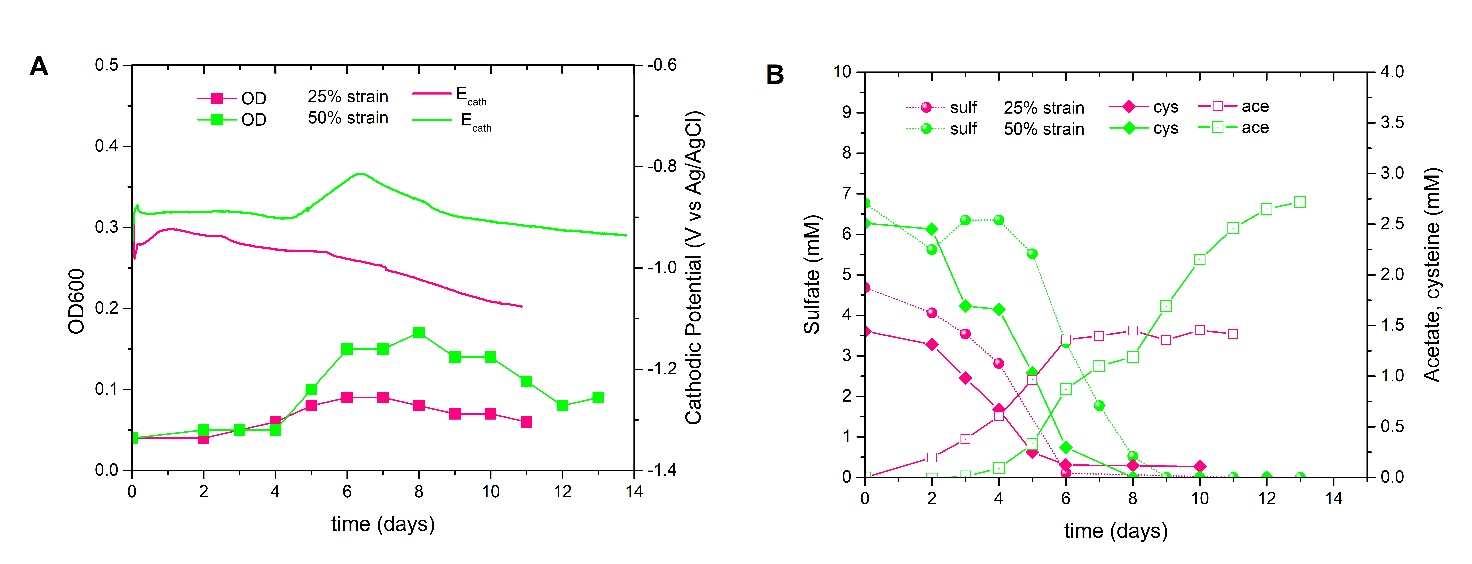


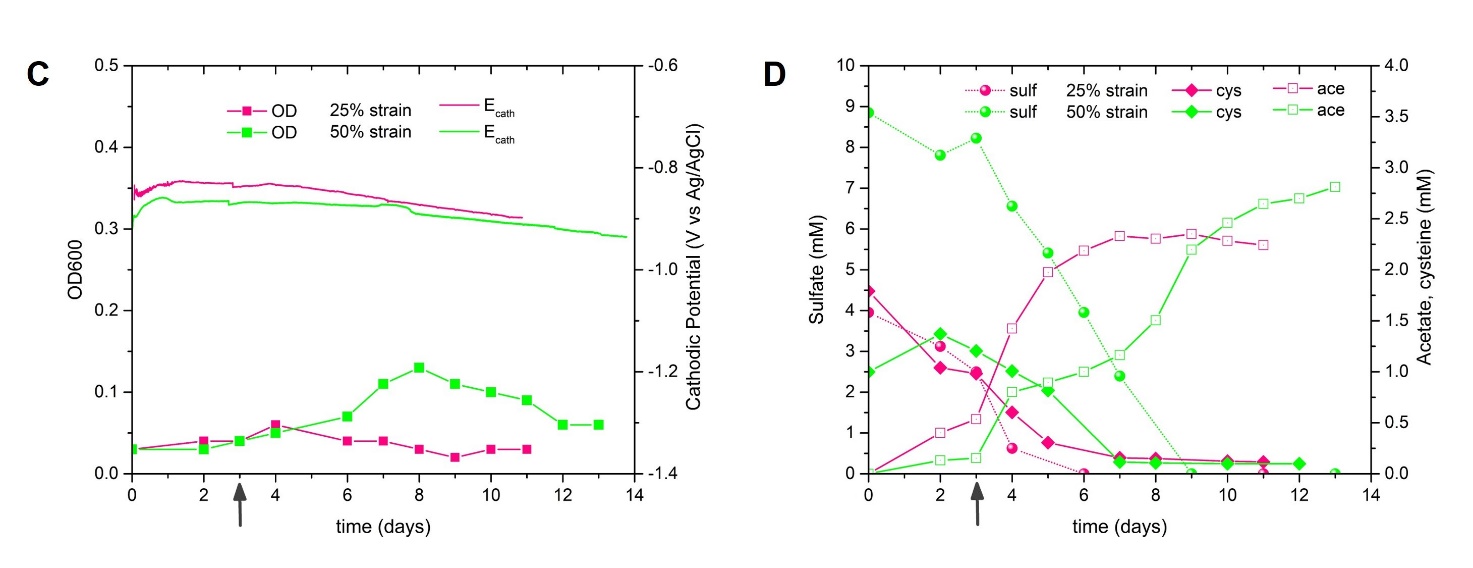


**Figure S6** Replicates of -15 mA Biocathodes inoculated with *D. orientis* adapted strains (of Figure 5 C and D and Figure 6). **(A)** OD and cathodic potential profiles and **(B)** sulfate reduction, cysteine consumption and acetate production profiles of 50% strains (13^th^ culture transfer as inoculum) and 25% strains (13^th^ culture transfer as inoculum). **(C)** OD and cathodic potential profiles replicates of experiments with bicarbonate spiking and **(D)** sulfate reduction, cysteine consumption and acetate production profiles of this experiment (compare to Figure 6). 50% sulfate biocathodes were inoculated with the 16^th^ culture transfer, while 25% sulfate biocathodes with the 15^th^ culture transfer. Dark grey arrows indicate the second pulse of 1 g L^1^ Na-bicarbonate.
